# The essential role of 2,4-dienoyl-CoA reductase for the degradation of complex fatty acid mixtures

**DOI:** 10.1128/mbio.02203-25

**Published:** 2025-10-29

**Authors:** Veronica Schiaffi, Viola Pavoncello, Bastien Prost, Audrey Solgadi, Frédéric Barras, Emmanuelle Bouveret

**Affiliations:** 1 SAMe Unit, Department of Microbiology, UMR CNRS 6047, Université Paris-Cité, Institut Pasteur 27058, Paris, France; 2 UMS-IPSIT–Platform SAMM, Université Paris-Saclay 27058, Orsay, France; National University of Singapore, Singapore, Singapore

**Keywords:** fatty acid degradation, *Escherichia coli*, [4Fe-4S] cluster, 2,4‑dienoyl‑CoA reductase, DECR, FadH, linoleic acid

## Abstract

Fatty acids (FAs) can be used as a carbon and energy source by most bacteria. FAs are diverse and show variations in aliphatic chain length, degree and kind of branching, and number of double bonds. After activation by a thioester link to coenzyme A (CoA), FAs are degraded by the β-oxidation machinery. The core β-oxidation enzymes can degrade most FAs, except those that bear an unsaturation at even-numbered carbons. Such FAs include the essential arachidonic and linoleic FAs of the mammalian diet. We studied the role of the 2,4-dienoyl-CoA reductase FadH in *E. coli* FA metabolism. We showed that *fadH* is essential for growth on linoleic acid and that cysteine residues connecting the FadH-bound [Fe-S] cluster are essential for activity *in vivo*. Eukaryotes also use 2,4-dienoyl-CoA reductases for β-oxidation in mitochondria, but these enzymes belong to a different family than FadH, with different co-factors and mechanism. Yet, we showed that eukaryotic 2,4-dienoyl-CoA reductases DECR can complement the *E. coli fadH* mutant for growth on linoleic acid. Moreover, we found that when mixed with other FAs, linoleic acid prevents the growth of the *fadH* mutant. This was shown to result from the jamming of the β-oxidation by unprocessed linoleic acid, and this blocking was relieved by producing FadH or eukaryotic DECRs. These studies demonstrate the key role of prokaryotic and eukaryotic 2,4-dienoyl-CoA reductases in complex environments containing mixtures of saturated and unsaturated FAs, and might pave the way to search for chemicals targeting DECR activity.

**IMPORTANCE:**

Bacteria and eukaryotes can harness energy from fatty acids (FAs) through the process of β-oxidation. However, information on the β-oxidation in bacteria stems from studies in which degradation of only a limited set of saturated or monounsaturated FAs was investigated, far from reflecting the wide chemical diversity of FAs found in nature. Here, we evidenced the physiological importance of dienoyl-CoA reductase enzymes required for the degradation of specific unsaturated FAs in complex mixtures of FAs, and how their absence leads to the congestion of the β-oxidation machinery. These results will permit a better understanding of the impact of FA degradation in enterobacteria, living in the complex gut environment where FAs are available from the diet or from host lipids. Furthermore, we showed that eukaryotic enzymes can replace the prokaryotic ones, opening the possibility of biomedical application in structure/function studies of the eukaryotic dienoyl-CoA reductases.

## INTRODUCTION

**Fig 1:**
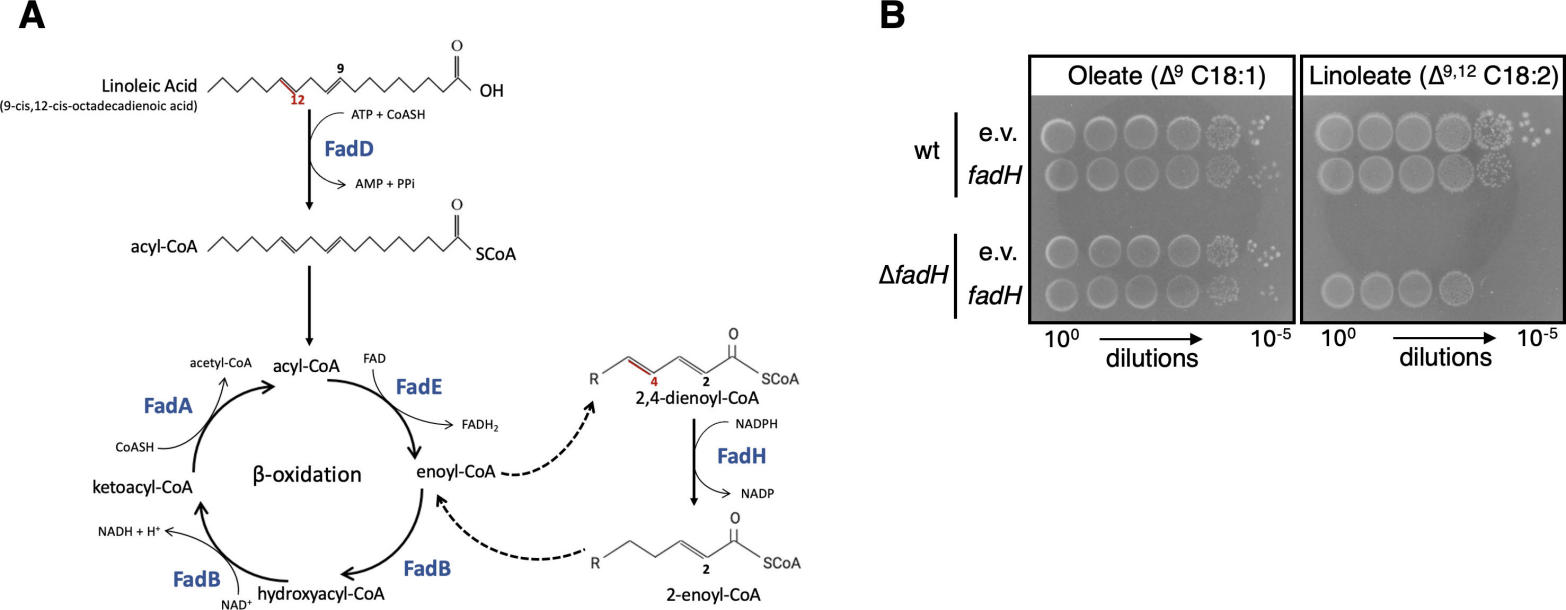
(**A**) Degradation of FAs in *E. coli*. After their import into the cell, FAs must be linked by a thioester to Coenzyme A to enter the β-oxidation cycle, an activity performed by the acyl-CoA synthase FadD. Each round of β-oxidation consists of the successive reactions of acyl-CoA dehydrogenase (FadE), enoyl-CoA hydratase (FadB), hydroxyacyl-CoA dehydrogenase (FadB), and finally 3-ketoacyl-CoA thiolase (FadA). Degradation of FAs with unsaturations at even-numbered carbon (indicated in red in the linoleic acid formula) leads to the production of a 2-*trans*,4-*cis* dienoyl-CoA intermediate. In the case of linoleic acid, this happens after 4 cycles of degradation, producing a 10-carbon-long 2,4-decadienoyl-CoA molecule. The 2,4-dienoyl-CoA reductase FadH takes reducing power from NADPH to catalyze the formation of an enoyl-CoA that can then re-enter the β-oxidation cycle at the enoyl-CoA hydratase step performed by FadB. (**B**) Wild-type (wt) and *fadH* mutant (FBE765) strains were transformed by pTrc99a or pTrc-*fadH* plasmids and tested for growth on oleate or linoleate as the sole carbon source as described in Materials and Methods. e.v. : empty pTrc vector.

Fatty acids (FAs) are present in the membranes of all organisms as building blocks of lipids, but they are also a potential source of carbon and energy. In bacteria, free FAs can arise either from the recycling of the lipids of the envelope or from being imported from the environment. In the gut, for example, free FAs can be released by the action of lipases on triglycerides from dietary fats, and numerous other examples of host-derived FAs abound in the literature (1). Degradation of FAs through β-oxidation leads to the production of acetyl-coenzyme A that can enter the TCA cycle, and to the production of reduced NADH and FADH_2_ cofactors (2–4) (Fig. 1A). Note that FAs can also display antimicrobial activities against which bacterial β-oxidation might play a detoxification role (5).

To be used by the β-oxidation cycle, free FAs are first linked as thioesters to coenzyme A (CoA) by the acyl-CoA synthetase FadD. Then, each round of β-oxidation consists of sequential activities of FadE, an acyl-CoA dehydrogenase, FadB, an enoyl-CoA hydratase and hydroxyacyl-CoA dehydrogenase, and FadA, a 3-ketoacyl-CoA thiolase (Fig. 1A). FadA and FadB form a so-called core trifunctional multienzyme complex, catalyzing the last three steps listed above. Noticeably, FadB also possesses 3,2-enoyl-CoA isomerase and 3-hydroxyacyl-CoA epimerase activities, which are both required for the degradation of unsaturated FAs (UFAs) with double bonds at odd-numbered positions.

The expression of the genes involved in the degradation of FAs (*fad* genes) is highly controlled. In the absence of exogenous FAs, *fad* genes are tightly repressed by the FadR transcriptional regulator. This repression is relieved by the binding of long-chain acyl-CoA to FadR, which happens when free FAs are available (2, 3). In *E. coli*, FadR can robustly detect only long-chain acyl-CoA of length 14 or more carbon long (6). Consequently, wild-type *E. coli* cannot grow on medium-chain FAs (MCFAs; 10–12 carbons long) as the sole carbon source simply because the β-oxidation machinery is not induced. In contrast, a ∆*fadR* mutant, in which the *fad* genes are constitutively expressed, can perfectly grow on MCFAs (7).

In addition to the core β-oxidation machinery described above, degradation of UFA with double bond*s* at even-numbered positions requires an additional enzyme. Indeed, degradation of such UFA leads to a 2-*trans*,4-*cis-*dienoyl-CoA intermediate that cannot be further catabolized by the core trifunctional enzymatic complex (Fig. 1A) (8). Biochemical investigations showed that *E. coli* produces a 2,4-dienoyl-CoA reductase, FadH, which can act on such UFAs, while a *fadH* mutant was found to preclude growth on petroselinic acid that contains a double bond at carbon 6 (9–11).

*In vitro*, FadH catalyzes the reduction of 2,4-dienoyl-CoA to 2-*trans*-enoyl-CoA. FadH is a 72 kDa monomeric protein (11) that contains several cofactors: NADPH, a [4Fe4S] cluster, a flavin adenine dinucleotide (FAD), and a flavin mononucleotide (FMN) (12, 13). FadH uses the reducing power from NADPH to remove the C_4_-C_5_ double bond and produce an enoyl-CoA that can re-enter the β-oxidation cycle (Fig. 1A) (10, 14). In the reduction process, two reducing equivalents are transferred from NADPH to FAD, then two electrons are further transferred to FMN via the [4Fe-4S] cluster (13, 15). The fully reduced FMN, together with the Tyr-166 and His-252 residues forming a catalytic dyad at the active center of the protein, performs the final reduction of the dienoyl-CoA substrate (13). Interestingly, FadH lacks stereospecificity and can catalyze the reduction of both *cis* and *trans* double bonds (16).

Like bacteria, eukaryotic cells use FAs as a source of energy through their β-oxidation in organelles. Recent studies highlighted the role of FA β-oxidation in the survival and proliferation of some types of cancer cells. In particular, a eukaryotic 2,4-dienoyl-CoA reductase (euDECR) was found to be induced in breast cancer and in treatment-resistant prostate cancer, correlating the gene to a higher malignancy and proliferation of the tumor (17, 18). Eukaryotic cells synthesize two 2,4-dienoyl-CoA reductases, a mitochondrial (euDECR1) and a peroxisomal (euDECR2) one (19–21). The mitochondrial and peroxisomal euDECRs carry out similar reactions, and they share 35% sequence identity (21). In contrast, they widely differ from the prokaryotic FadH. The euDECRs are homotetramers with a total molecular mass of 124 kDa. They are strictly NADPH-dependent, but differ from FadH by lacking FAD, FMN, and [4Fe-4S] cofactors. In euDECR, the reducing equivalents are directly supplied from NADPH to the substrate to form 3-*trans*-enoyl-CoA (14). Then, an additional isomerase is required to convert the 3-*trans*-enoyl-CoA to 2-*trans*-enoyl-CoA, which can reenter the β-oxidation cycle. The euDECR can also metabolize FAs with double bonds at odd-numbered positions (11, 22), whereas there is no evidence for such activity in the *E. coli* FadH.

In the present study, we show that *fadH* is essential for *E. coli* to degrade linoleic acid (9-*cis,* 12-*cis-*octadecadienoic acid). Moreover, we demonstrate that FadH activity is essential to prevent linoleic acid degradation products from jamming the β-oxidation and thereby inhibiting the degradation of other types of FAs. An extension of our study to eukaryotic 2,4-dienoyl-CoA reductases shows that they can substitute for FadH in *E. coli*, both for growth on linoleate and preventing from inhibition of other FA degradation. Our results highlight the crucial role that enoyl-CoA reductases may play in the degradation of FAs in natural environments, either for the use of FAs as energy or for detoxification of host-derived lipids with antimicrobial effects (5).

## RESULTS

### *fadH* is essential for the growth of *E. coli* on linoleic acid

We compared the growth of a *fadH* mutant with the wild-type strain on minimal medium containing oleate (double bond at the odd C9 carbon position) or linoleate (double bond at the even C12 carbon position) as the sole carbon and energy source (Fig. 1B). Growth of the wild-type strain appeared as robust on linoleate as on oleate. In contrast, the *fadH* mutant was unable to grow on linoleate, whereas it was able to grow on oleate. Growth on linoleate of the *fadH* mutant was restored when the mutant strain was complemented with a pTrc-*fadH* plasmid (Fig. 1B). Note that this complementation was observed in the absence of induction with IPTG, showing that low amounts of *fadH* expression from the leaky Ptrc promoter were sufficient for complementation.

### The [Fe-S] cluster of FadH is required for FadH activity *in vivo*

FadH binds a [4Fe-4S] cluster, predicted to be liganded by four cysteine residues (Cys335, Cys338, Cys342, and Cys354) as shown by structural analysis (13). Purified FadH variants with a Cys338Ala or a double Cys335Ala-Cys338Ala mutation have drastically reduced activity *in vitro* (15). Therefore, we tested the importance of the two remaining Cys342 and Cys354 residues. We mutated each of the 4 cysteine residues to alanine in the pTrc-*fadH* plasmid. We first verified that the mutant proteins were produced similarly to the wild-type FadH protein when overproduced with 1 mM IPTG (Fig. S1A, left panel). We also used a pTrc-*fadH-*SPA plasmid enabling us to specifically detect the FadH protein by western blot, even in the absence of IPTG (Fig. S1A, right panel).

**Fig 2:**
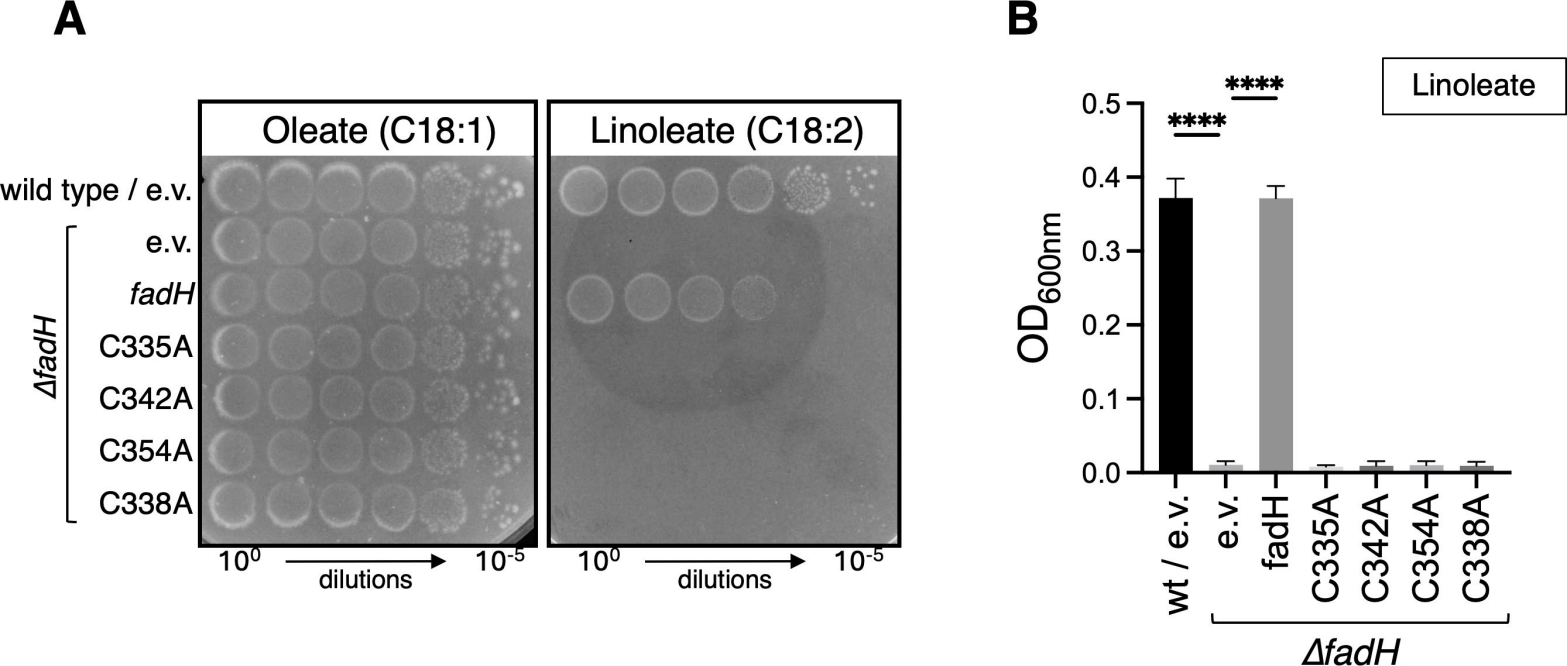
The *fadH* deletion strain (FBE765) was transformed by pTrc99a empty vector (e.v.), pTrc-*fadH* (**A**), or pTrc-*fadH*-SPA (**B**), and plasmids encoding *fadH* containing point mutations replacing the indicated cysteine residues by alanines. The transformed strains were tested for growth on oleate or linoleate as a sole carbon source either on agar plates (**A**) or in liquid (**B**) as described in Materials and Methods. We performed ordinary one-way ANOVA statistical tests for comparison with the ∆*fadH*/e.v. condition using Prism software. ****P < 0.0001; bars without display showed no significant difference (*P* > 0.05).

We then tested the ability of the cysteine mutants to complement the growth phenotype of ∆*fadH* on linoleate plates (Fig. 2A) or in liquid cultures with linoleate as the sole carbon source (Fig. 2B). When expressed from plasmids, none of the mutants enabled complementation. Our results show that Cys335, Cys338, Cys342, and Cys354 are all essential for FadH function *in vivo*, and by inference, that the [4Fe-4S] cluster is essential for FadH activity *in vivo*.

### The unsaturated linoleic acid inhibits growth of the *∆fadH* mutant on other FAs

**Fig 3:**
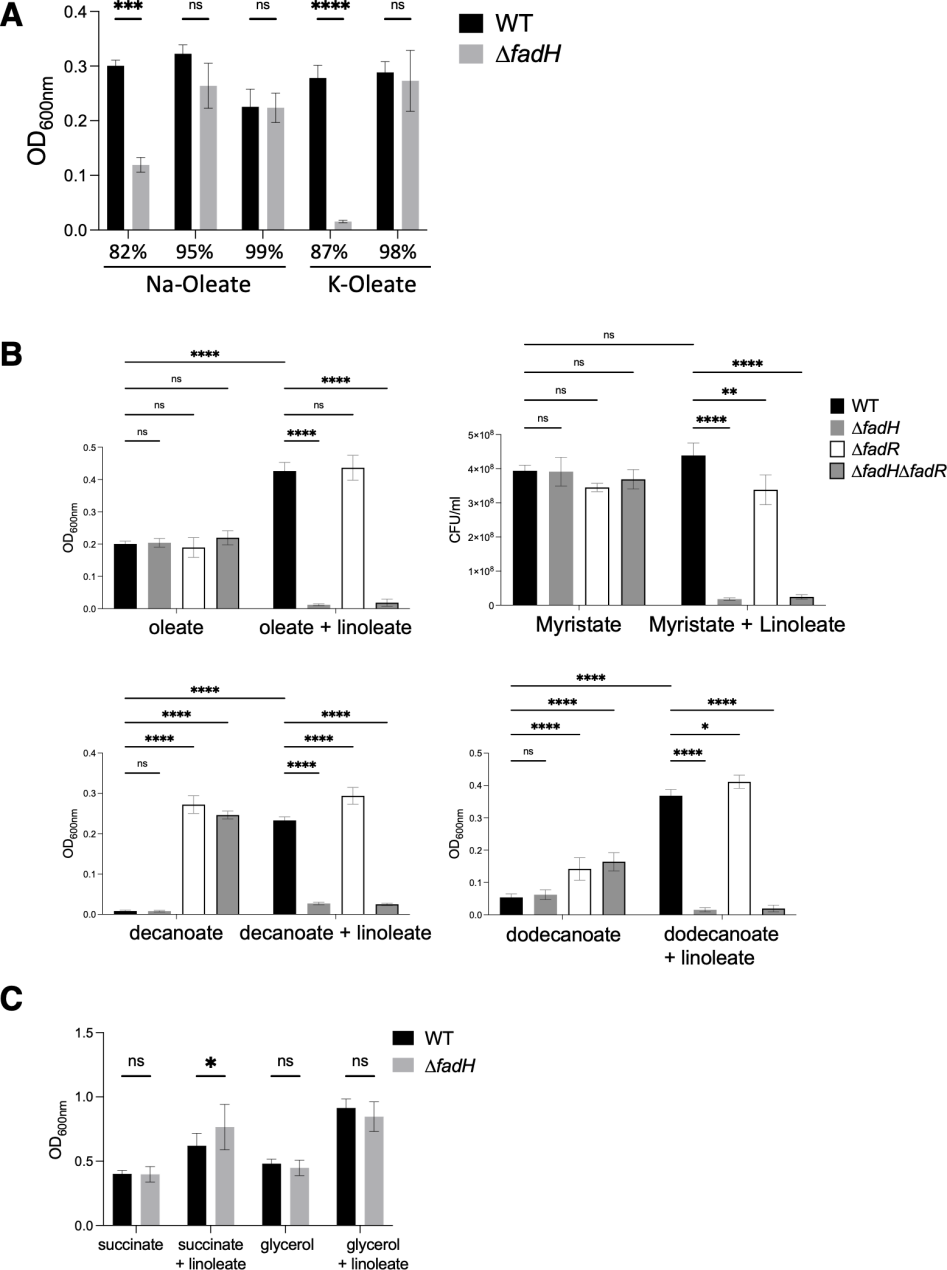
(**A**) Liquid growth of wild-type and *fadH* mutant (FBE765) strains was tested in minimal medium containing the following commercial sources of oleate: Na-oleate 82% (Sigma#26125); Na-oleate 95% (Sigma#O3880); Na-oleate 99% (Sigma#O7501); K-oleate 87% (Sigma#60420); and K-oleate 98% (TCI#O0056). The graph presents the mean final OD_600_ of 3 replicates after 48 hours of incubation at 37°C as described in Materials and Methods. (**B**) Liquid growth of wild-type, *fadH* (FBE765), *fadR* (FBE189), and ∆*fadH*∆*fadR* (FBE1197) mutant strains was tested in minimal medium containing FA (0.1% K-oleate [TCI], myristate, decanoate, or dodecanoate), with or without the addition of 0.1% linoleate as indicated. (**C**) Liquid growth of wild-type and *fadH* mutant (FBE765) strains was tested in minimal medium containing 0.4% succinate or 0.2% glycerol, with or without the addition of 0.1% linoleate as indicated. For B and C, the graphs present the mean final OD_600_ of 6 replicates after 48 hours of incubation at 37°C as described in Materials and Methods. For growth in myristate, the graph presents the mean colony-forming units (cfu) of 4 replicates. The error bars depict the standard deviation. We performed systematic two-way ANOVA statistical tests using Prism software. ns: not significant (*P* > 0.05); *P = 0.036.

During the course of this project, we tested the growth of *E. coli* on several commercial sources of oleate. Surprisingly, the *fadH* mutant showed clear growth defect when using powders rated less than 90% pure, whereas it exhibited wild-type-like growth on pure oleate preparations rated more than 95% (Fig. 3A). Changing the type of neutralization salt (sodium or potassium) did not modify the growth defect phenotype (Fig. 3A). Given the results above revealing the requirement of FadH to grow on UFA with double bonds at even-numbered carbon positions, we surmised that such type of UFA may be contaminating the oleate preparations. We tested this hypothesis by mixing linoleate with high-quality oleate powder. Indeed, the presence of linoleate prevented the use of pure oleate by the ∆*fadH* mutant (Fig. 3B; Fig. S2A). A possibility was that the presence of linoleate or other types of UFAs somehow inhibited FA degradation as a whole by blocking β-oxidation in the ∆*fadH* mutant. If so, the prediction was that the presence of linoleate should prevent the use of any kind of FAs by the *fadH* mutant, but not the use of other types of carbon source. Consistent with this prediction, the ∆*fadH* mutant also failed to grow on a mix of linoleate and myristate (a 14-carbon saturated FA) (Fig. 3B; Fig. S2A).

Then, to test the linoleate inhibitory effect on MCFAs, such as decanoate (C10:0) and dodecanoate (C12:0), we had to use a ∆*fadR*∆*fadH* double mutant. Indeed, as explained in the introduction section, wild-type *E. coli* normally cannot grow on decanoate or dodecanoate (since the FadR repressor does not respond to FAs with chain length under 14 carbon long), but a ∆*fadR* mutant (in which the *fad* genes are constitutively expressed) is able to grow on these MCFAs (Fig. 3B; Fig. S2B). So, while the ∆*fadR*∆*fadH* mutant grew well on decanoate or dodecanoate, it was unable to grow when linoleate was added (Fig. 3B; Fig. S2B).

Last, we tested whether linoleate was inhibiting the use of carbon sources that were not FAs. This proved not to be the case as growth of the ∆*fadH* strain on media containing succinate or glycerol as carbon sources was not affected by the presence of linoleate (Fig. 3C; Fig. S2C). This showed that linoleate was inhibiting use of FAs specifically.

### FAs accumulate inside *∆fadH* mutant cells exposed to linoleate

A possible explanation to account for the linoleate-mediated inhibition of FA utilization in a ∆*fadH* mutant was that the Fad machinery got jammed by unprocessed products of linoleic acid degradation. This was tested in three ways.

**Fig 4:**
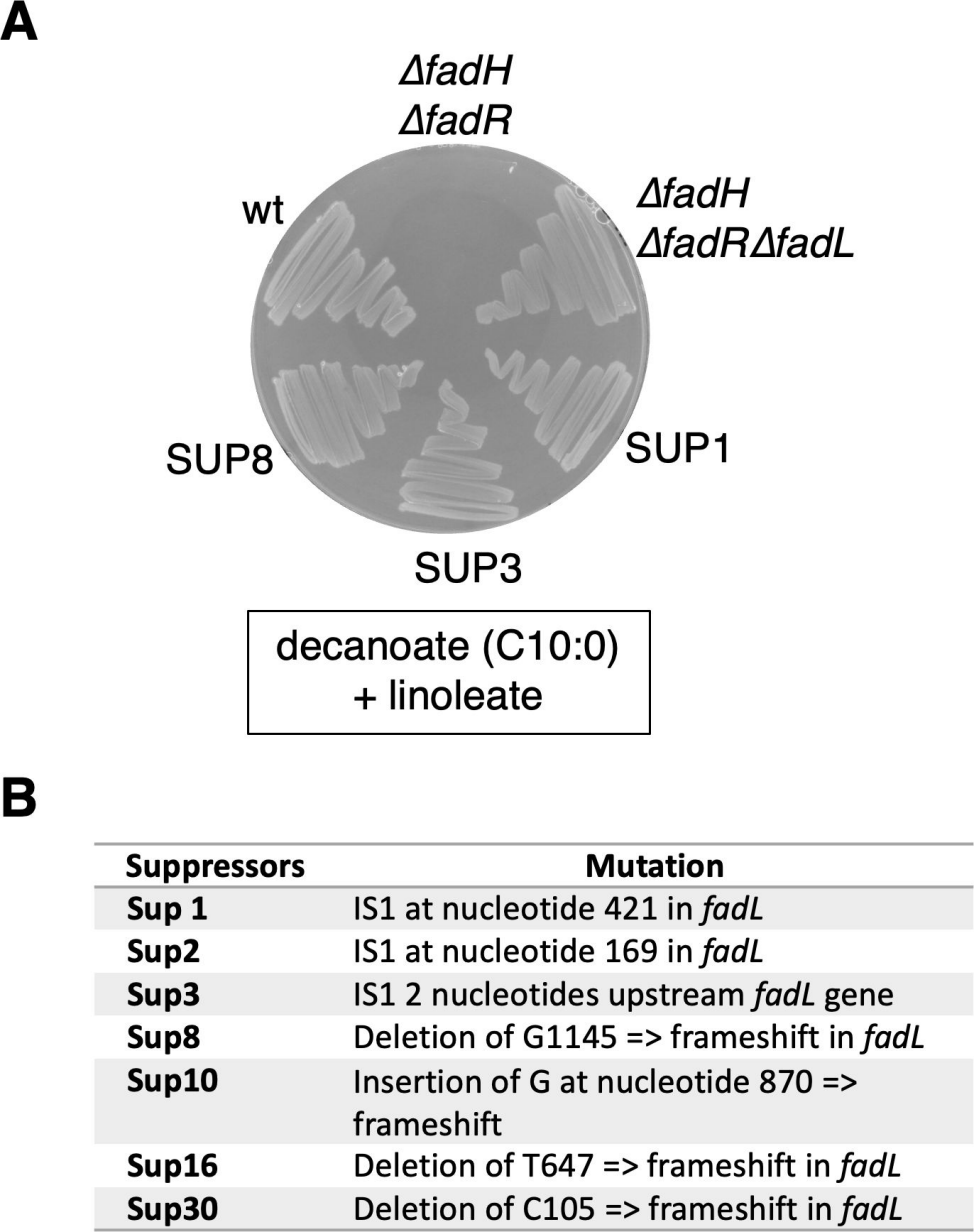
(**A**) The wild-type, ∆*fadH* (FBE765), ∆*fadH*∆*fadR* (FBE1197), ∆*fadH*∆*fadR*∆*fadL* (VSS50), and three suppressor strains (SUP1, SUP3, and SUP8) derived from the ∆*fadH*∆*fadR* strain were isolated on M9 minimal medium plates containing 0.1% decanoate and 0.1% linoleate and incubated for 3 days at 37°C. (**B**) *fadL* mutations in the suppressors of the ∆*fadH*∆*fadR* strain for growth on decanoate + linoleate.

First, we reasoned that preventing the entrance of linoleic acid inside the cell might prevent the jamming of the machinery. Long-chain FAs such as linoleic acid are imported via the outer membrane transporter FadL. Therefore, we tested whether a ∆*fadL* mutation would suppress the growth defect of the ∆*fadH*∆*fadR* strain on linoleate added with decanoate (a 10-carbon FA that itself does not require FadL for transport), a condition for which growth inhibition was the strongest. The ∆*fadL* mutation was transduced in the ∆*fadH*∆*fadR* strain, and the resulting transductants were able to grow on decanoate even in the presence of linoleate (Fig. 4A), demonstrating that the linoleate inhibitory effect required linoleic acid to be imported inside the cells.

Second, we searched for suppression mutations in the ∆*fadH*∆*fadR* strain plated on media with linoleate and decanoate. We obtained suppressors that acquired the capability to grow in this condition (Fig. 4A). Most of the suppressors had an inactivated *fadL* gene, either by the insertion of an IS1 element or by nucleotide deletions inducing frameshifts inside the *fadL* ORF (Fig. 4B). To avoid selection of suppressors with mutations in *fadL*, we performed an additional screen on linoleate added to myristate this time (requiring a functional FadL transporter for growth). We obtained one stable suppressor whose whole-genome sequencing uncovered a duplication of the 100 kb large genomic region between the ribosomal RNA operons *rssA* and *rssC*. This region includes many genes potentially related to FA metabolism, such as *yigI*, *pldA*, and *pldB* (thioesterase and lipases). The molecular basis of their suppressor effect will be investigated in a separate study.

**Fig 5:**
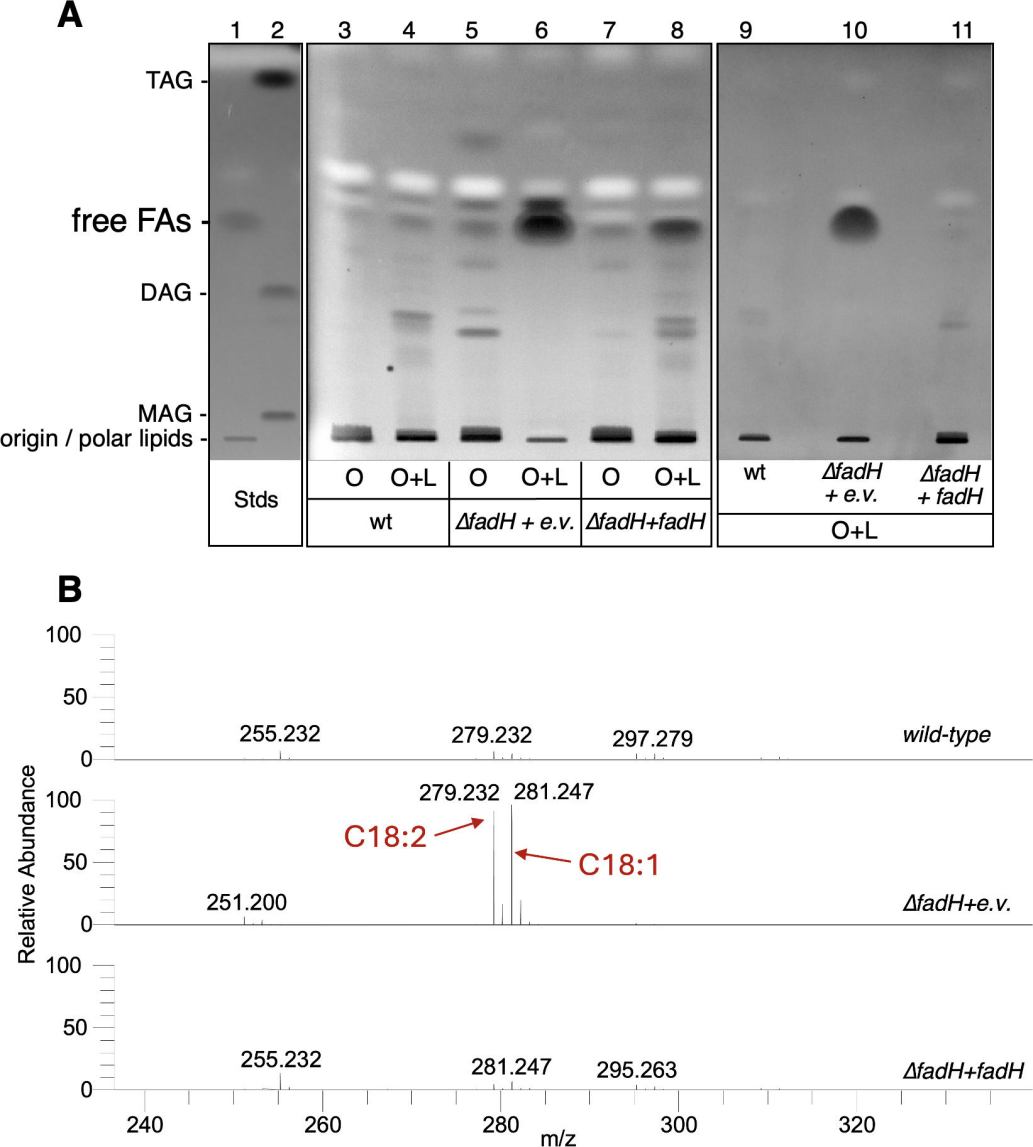
(**A**) Total lipids were extracted from cultures of wild-type (wt) and ∆*fadH* (FBE765) strains transformed with pTrc empty vector (e.v.) or pTrc-*fadH (fadH)*, grown in M9 minimal medium containing oleate (**O**) or containing oleate plus linoleate (O + L) (see Materials and Methods for the exact growth conditions). Neutral lipids were analyzed by TLC and detected with primuline staining as described in Materials and Methods. Lanes 1 and 2 show the standards (Stds) for the neutral lipid TLC: Lane 1: Free FAs (lipid extract from the ∆*fadD* strain). Lane 2: standard mono (MAG), di (DAG), and triglycerides (TAG). Lanes 9, 10, and 11 correspond to a separate preparation of lipid extracts from cultures in oleate plus oleate, prepared for lipidomic analysis. (**B**) Mass spectra of the free FA fractions of the three strains grown in oleate plus linoleate (lanes 9–11 of panel A) with the same scale detected with negative atmospheric pressure chemical ionization (APCI). The C18:1 (m/z 281.247) and C18:2 (*m*/*z* 279.232) peaks correspond to oleic and linoleic acids, respectively.

Third, total lipid content from wild-type and ∆*fadH* strains was analyzed by both thin-layer chromatography (TLC) and lipidomics. Both strains were grown in minimal medium with oleate as the sole carbon source for 2 days, then these cultures were diluted in minimal medium containing oleate with or without linoleate and incubated for two more days. Note that the growth of the ∆*fadH* strain was strongly affected by linoleate + oleate as described above, but we could obtain enough cell material in these growth conditions with a preincubation with oleate (see Materials and Methods for precise growth conditions). TLC analysis of the neutral lipids revealed accumulation of free FAs inside the cells in the ∆*fadH* mutant exposed to linoleate (Fig. 5A). When the ∆*fadH* strain was transformed with the pTrc-*fadH* plasmid, liquid growth in the presence of linoleate and oleate was restored and similar to the growth of the wild-type strain. Depending on the experiment, this was concomitant with a strong decrease or even a complete disappearance of the endogenous free FAs (Fig. 5A, lane 8 compared to lane 6 or lane 11 compared to lane 10). To identify the exact nature of the free FAs accumulating inside the cell in the ∆*fadH* mutant in the presence of oleate and linoleate, we performed a global lipidomic analysis (see Materials and Methods). Mass spectrometry analysis of the free FA fraction identified the full-length oleic and linoleic acids as the predominant FA species accumulating in the ∆*fadH* mutant (Fig. 5B). Taken together, these results strongly suggest that linoleate exerts its inhibitory effect in the ∆*fadH* mutant by blocking the β-oxidation machinery.

### Linoleic acid jams the β-oxidation in a ∆*fadH* mutant

To investigate the basis of the linoleate-mediated inhibitory effect on oleate degradation, we tested whether there could be actual competition between the two FAs. Supporting this view, we observed that the extent of growth inhibition of the ∆*fadH* mutant was proportional to the concentration of linoleate present in the linoleate/oleate mix (Fig. S3). A logical follow-up was that linoleate inhibited oleate degradation by outcompeting FA degradation machinery components. In addition, the linoleate inhibitory effect on oleate degradation was not as strong in the ∆*fadR*∆*fadH* mutant as in the ∆*fadH* mutant, as observed on agar plates (Fig. S2A). Because the ∆*fadR* mutation turns on constitutive synthesis of the Fad degradation machinery, this suggests that the jamming of the machinery could be suppressed by increasing the levels of the Fad proteins for which oleic acid and linoleic acid were competing. Note, however, that we could not observe this partial suppression by ∆*fadR* in liquid growth (compare Fig. 3 and Fig. S2A), and this was only observed for growth on oleate + linoleate (not on smaller FAs + linoleate).

**Fig 6:**
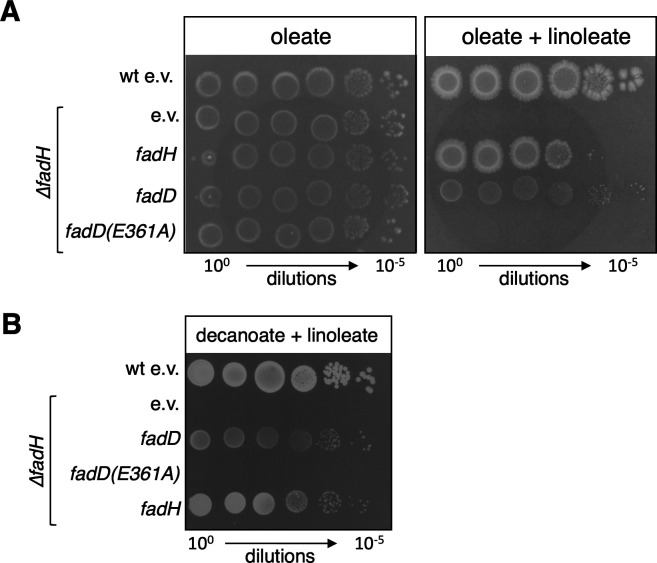
The *fadH* deletion strain (FBE765) was transformed by pTrc99a empty vector (e.v.), and plasmids expressing *fadH*, *fadD*, or *fadD*(E361A) inactive mutant. The transformed strains were tested for growth on minimal medium containing oleate, oleate plus linoleate (**A**), or decanoate + linoleate (**B**) as described in Materials and Methods.

We then tested whether overexpression of *fad* genes could act as multicopy suppressors of the linoleate inhibitory effect in the ∆*fadH* mutant. We found that the overproduction of the acyl-CoA synthetase FadD improved the growth of the ∆*fadH* mutant on the oleate/linoleate mix (Fig. 6A and S4A) or on the decanoate/linoleate mix (Fig. 6B) on solid media. None of the other Fad enzymes improved growth on linoleate + oleate (Fig. S4A). To test whether suppression by increased *fadD* gene dosage was due to acyl-CoA synthase activity of the FadD enzyme, we constructed the Y213A and E361A variants. These residues are located in the ATP/AMP signature motif, and the corresponding FadD mutated enzymes retain only 10% or less of wild-type activity *in vitro* (23). The cognate alleles were introduced in the pTrc-*fadD* plasmid. As we have done previously for FadH, we also constructed a pTrc-*fadD-*SPA plasmid to verify the production of the FadD-mutated proteins similar to the wild-type FadD protein (Fig. S1B). *In vivo*, we observed that only the FadD(E361A) mutant was severely impaired in FadD activity (Fig. S4B and C); therefore, we selected this mutant for further experiments. Compared to wild-type FadD, the FadD(E361A) mutant was not able to restore growth on linoleate + oleate or on linoleate + decanoate in the ∆*fadH* mutant (Fig. 6A and B), showing that the enzymatic activity of FadD was required to relieve the jamming of the FA degradation machinery by linoleate. All together, these experiments demonstrated that the *fadH* gene is essential for *E. coli* to use FA optimally in a complex environment containing mixtures of FA and UFA, a situation likely to reflect one found in the gut.

### Petroselinic acid also jams FA β-oxidation in a ∆*fadH* mutant

**Fig 7:**
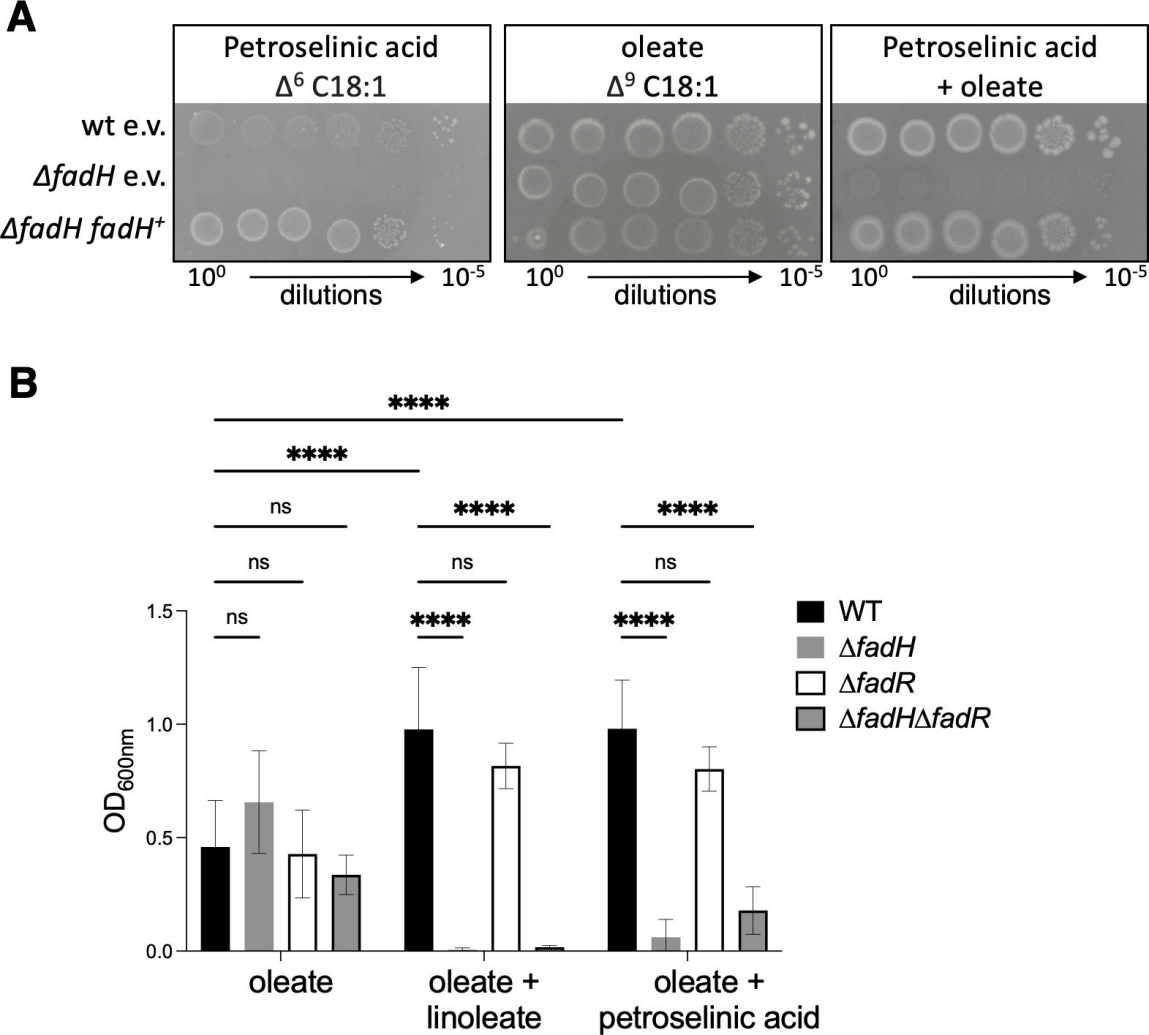
(**A**) Wild-type (wt) and ∆*fadH* mutant (FBE765) strains were transformed by pTrc99a empty vector (e.v.) or pTrc-*fadH* plasmids and tested for growth on petroselinic acid and oleate with or without petroselinic acid as the sole carbon source as described in Materials and Methods. Note that the middle panel is a copy of the first three rows of the left panel of Fig. 6A. (**B**) Wild-type (wt), ∆*fadH* (FBE765), ∆*fadR* (FBE189), and ∆*fadH*∆*fadR* (FBE1197) mutant strains were tested for liquid growth in minimal medium containing 0.1% oleate alone, oleate and 0.1% linoleate, or oleate and 0.1% petroselinic acid, as described in Materials and Methods. The graph presents the mean final OD_600_ of 6 replicates after 48 hours of incubation at 37°C as described in Materials and Methods. The error bars depict the standard deviation. We performed systematic two-way ANOVA statistical tests using Prism software. ns : not significant (*P* > 0.05).

Similar to the results presented here showing that *fadH* is required for growth on linoleate as the sole carbon source, it was previously shown that *fadH* is required for degradation of petroselinic acid (∆^6^ C18:1), an unsaturated FA containing a unique double bond at the even C6 carbon position (10). Therefore, we set up to test whether the presence of petroselinic acid also prevented the use of other FAs by the ∆*fadH* mutant (Fig. 7). As expected, a ∆*fadH* mutant was unable to grow on petroselinic acid alone, while the complemented strain with the pTrc-*fadH* plasmid grew well (Fig. 7A, left panel). When both oleate and petroselinic acid were present, the ∆*fadH* mutant was unable to use oleate for growth, while the wild-type and ∆*fadH*/pTrc-*fadH* complemented grew fine (Fig. 7A, right panel). Identical results were obtained when testing the growth of the same strains in liquid minimal medium (Fig. 7B). This result strongly supports the notion that FadH is required to prevent the FA degradation machinery from being blocked in the presence of any type of FA containing a double bond at an even position.

### Eukaryotic 2,4-dienoyl-CoA reductases (euDECR) can complement an *E. coli* ∆*fadH* mutant

Given that β-oxidation is highly conserved between prokaryotes and eukaryotes, it was intriguing that eukaryotes make use of a 2,4-dienoyl-CoA reductase that exhibits structural and functional differences with FadH, especially the absence of an [Fe-S] cluster in euDECR, and a different product (see Introduction).

**Fig 8:**
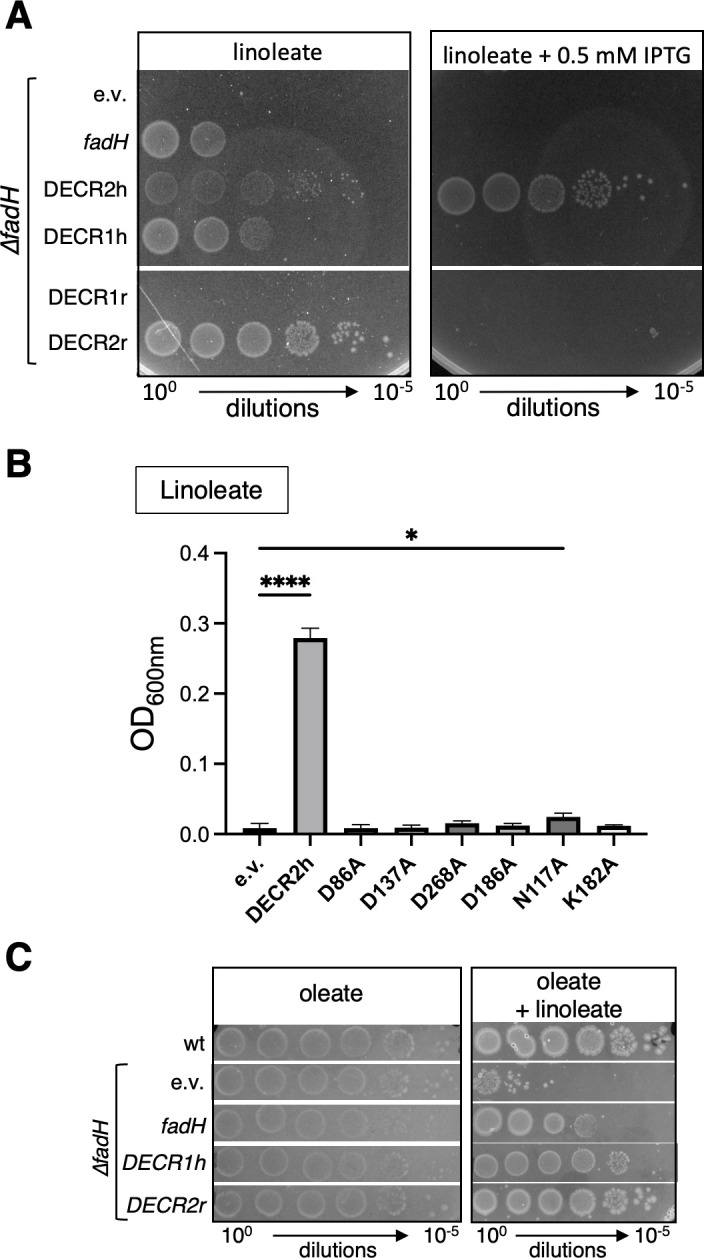
(**A**) The *fadH* deletion strain (FBE765) was transformed by pTrc99a empty vector (e.v.), *fadH* vector, or plasmids encoding the indicated eukaryote DECR constructions. The transformed strains were tested for growth on linoleate, with or without 0.5 mM IPTG, as described in Materials and Methods. Each panel shows a picture taken from one plate; the white lines indicate a horizontal cut in the image to show different parts of the same plate. (**B**) The *fadH* deletion strain (FBE765) was transformed by pTrc99a empty vector (e.v.), pTrc-DECR2h plasmid, or the plasmids encoding the indicated mutated DECR2h genes. Liquid growth in minimal medium containing linoleate as the sole carbon source was assayed as described in the Materials and Methods. The graph presents the mean final OD_600_ of 4 replicates after 48 hours of incubation at 37°C as described in Materials and Methods. The error bars depict the standard deviation. We performed ordinary one-way ANOVA statistical tests for comparison with the e.v. condition using Prism software. *P = 0.017; ****P < 0.0001; the other conditions were not significant (*P* > 0.05). (**C**) The wild-type and ∆*fadH* mutant (FBE765) strains were transformed by the pTrc99a empty vector (e.v.) or by the pTrc plasmids expressing *fadH*, DECR1h, or DECR2r genes as indicated. The transformed strains were tested for growth on 0.1% oleate, with or without 0.1% linoleate, as described in Materials and Methods. Each panel shows a picture taken from one plate; the horizontal white lines indicate a cut to show different parts of the same plate.

Therefore, we tested whether euDECRs could complement an *E. coli fadH* mutant. We chose to test both mitochondrial DECR1 and peroxisomal DECR2 enzymes. We also tested both the human and the rat homologs (20, 24). DECR sequences (deleted of the transit peptide sequence in the case of mitochondrial DECR1 genes) were synthesized and codon-optimized for production in *E. coli*. For human DECR2, we used the original cDNA sequence that has been cloned before (20). Then, the sequences were cloned into the pTrc99a vector. The ∆*fadH* mutant was transformed by the DECR-expressing plasmids, and growth on linoleate was assayed using different concentrations of IPTG inducer (Fig. 8A; Fig. S5). Different complementation behaviors were observed depending on the DECR constructs. Human DECR1 and rat DECR2 were able to complement for the absence of FadH without induction, hence with basal expression from the Ptrc promoter. Strikingly, this complementation was lost when induced with IPTG, suggesting a toxic effect of the overexpression. This is similar to the expression of *fadH* itself that complemented the ∆*fadH* mutant without induction, but which was toxic when induced at 0.5 mM IPTG (Fig. 8A; Fig. S5). In contrast, the human DECR2 was only able to complement upon induction at IPTG concentrations higher than 0.01 mM IPTG. Finally, we did not observe complementation with the rat DECR1 construct in any condition. To test whether these differences in complementation were due to functional specificities or from a difference in protein production levels, we tested the production of the different DECR proteins, either with (Fig. S1D, left panel) or without IPTG, using an additional set of plasmids containing a SPA-tagged version of the proteins (Fig. S1D, right panel). Interestingly, for the human DECR2h-SPA protein, induction with 0.5 mM IPTG was required to obtain amounts comparable to the other SPA-tagged proteins obtained without induction (Fig. S1D, right panel). Overproduced human DECR1 and rat DECR2 proteins were easily identified on a Coomassie blue-stained gel, similarly to FadH. In conclusion, except for the rat DECR1, which was likely not functional, the differences in complementation behavior appeared to be due to different efficiencies of production of the proteins.

Next, we tested whether the use of *E. coli* could help to investigate functional aspects of eukaryotic DECR. As a proof of concept, we mutagenized residues of human DECR2 that have been proposed to be involved in its enzymatic activity (21) and tested their ability to complement the *E. coli* ∆*fadH* mutant for growth on linoleate. Mutations of acidic (D86, D137, D186, and D268) and conserved K182 residues around the active site abolished the complementation (Fig. 8B; Fig. S6A). Mutation of the conserved N117 residue retained a very weak level of complementation upon overproduction as detected on plates but barely visible in liquid cultures (Fig. 8A; Fig. S6A).

Finally, we asked whether the activity conferred by euDECR enzymes was able to relieve the jamming of the β-oxidation machinery by linoleate in the ∆*fadH* mutant. We chose the euDECR1h and euDECR2r plasmids that complemented the ∆*fadH* mutant for growth on linoleate alone without the need to add IPTG, like the pTrc-*fadH* plasmid. DECR2r and DECR1h (yet somewhat less efficiently) were able to relieve the inhibition of oleate degradation by linoleate in the ∆*fadH* mutant, both on solid and liquid media (Fig. 8C; Fig. S6B).

These results show that 2,4-dienoyl-CoA reductases from bacteria and eukaryotes are indeed able to perform the same reaction *in vivo* in *E. coli*. Interestingly, it suggests that in *E. coli*, an isomerase activity distinct from FadH is present to enable conversion from 3-*trans-*enoyl-CoA produced by euDECR to 2-*trans-*enoyl-CoA. Finally, these results of *in vivo* complementation of an *E. coli fadH* mutant may provide a practical and powerful tool to study the structure/function of the eukaryotic enzymes important for human health.

## DISCUSSION

In the gut, enterobacteria can encounter a mix of diverse FAs, which they can use as a source of energy for growth. Noticeably, recent evidence showed that cancer cells also rely heavily on FA metabolism for their growth and proliferation (25). In contrast, host-derived FAs can also display antimicrobial activities against which bacterial β-oxidation might play a detoxification role (5). Here, we demonstrated that the 2,4-dienoyl-CoA reductase FadH is essential for *E. coli* to use linoleic acid as the sole carbon and energy source. Moreover, we showed that FadH is essential for preventing jamming of the FA degradation machinery in a situation wherein *E. coli* is exposed to mixed heterogeneous environments, including saturated and (poly)unsaturated FAs, a situation likely to reflect natural conditions. Strikingly, we showed that eukaryotic DECR enzymes that show wide structural and biochemical differences from FadH can complement a ∆*fadH* mutant. Given the implication of euDECR enzymes in human health, especially some types of cancer (17, 26), this result opens the way to study euDECR structure/function in *E. coli* and eventually offers a practical tool for screening of anti-euDECR inhibitors.

In this work, we observed that FadH is essential for *E. coli* to use linoleic acid (and petroselinic acid) as an energy source. Quite strikingly, we showed here that FadH is also required for precluding these UFAs from inhibiting the degradation of any type of FAs. We showed that this inhibitory effect is due to the accumulation of intermediates of linoleic acid degradation that are likely to set an inhibitory competition situation. This hypothesis was supported by the fact that increasing amounts of the machinery by deleting the *fadR* repressor relieved the jam to some extent (Fig. S2A). Moreover, this result indicated that the *fad* genes were not completely derepressed in the presence of oleate and linoleate.

Interestingly, while increasing the expression of the *fad* genes in the ∆*fadH*∆*fadR* mutant improved degradation of oleate in the presence of linoleate, we did not observe any improvement in the degradation of myristate, decanoate, or dodecanoate (Fig. S2A and B). This suggests that the longer-chain oleate was better at displacing and competing with the stalled linoleic acid degradation product intermediates, possibly due to a higher specificity of the enzymes of the FA degradation machinery for long-chain FAs. In addition, we observed that increasing the levels of acyl-CoA inside the cell by overproducing FadD was also able to relieve the inhibition of the FA degradation machinery by linoleate in the ∆*fadH* mutant (Fig. 6; Fig. S4A). We propose two explanations for this. The first one is that increased acyl-CoA levels might further derepress the *fad* genes. In support of this hypothesis, it has been reported that overproduction of FadD increased the expression of the *fadE* gene and that it even permitted the cell to grow on MCFAs (27). However, as underlined above, increasing the expression of the *fad* genes in the ∆*fadH*∆*fadR* mutant was not enough to allow the consumption of decanoate in the presence of linoleate, while FadD overproduction did (Fig. 6B). Therefore, the second hypothesis is that the positive effect of FadD might be explained rather by an increased activity of the β-oxidation enzymes due to the higher acyl-CoA levels rather than the upregulation of the *fad* genes.

Linoleic acid is an 18-carbon-long UFA with two double bonds at carbons 9 and 12 (Fig. 1). Thus, in theory, four cycles of partial degradation of linoleic acid should be possible before the degradation process reaches the 10-carbon-long intermediate step requiring the FadH activity. Therefore, it could have been expected that a ∆*fadH* mutant might still be able to grow on the energy given by this partial degradation. However, the jamming that we describe here explains why it is not the case, as the machinery is completely blocked and the full-length FAs accumulate inside the cell. Any FA containing an unsaturation at an even-numbered carbon is expected to show the same behavior, as we observed with petroselinic acid. In addition to linoleic acid, which is essential and can be obtained only from the diet, most polyunsaturated FAs found in vegetable oils contain unsaturation at an even position. The results we obtained with low-quality oleate powders showed that even small amounts of polyunsaturated FAs can block β-oxidation in a ∆*fadH* mutant, suggesting an essential role of *fadH* in the complex and heterogeneous environment of the gut.

Interestingly, the 2,4-dienoyl-CoA reductase activity can be performed by two distinct protein families, the bacterial FadH-like containing a [4Fe-4S] cluster and the eukaryotic protein family lacking such a cofactor. We showed here that all four cysteine residues binding the cluster in FadH are required for *in vivo* activity and strongly support the notion of the essential role of the cluster for the reductase activity. The question of why eukaryotes have selected a reductase that functions without a [Fe-S] cluster is open. One possibility is that [Fe-S] independent 2,4-dienoyl-CoA reductases are more resistant to oxidative stress in the mitochondrial environment. Such an argument has been used for other types of enzymes, such as fumarases, to strengthen the idea that aerobic organisms have steadily evolved to get rid of enzymes requiring [4Fe-4S] clusters that are sensitive to oxidation (28). Yet, the [4Fe-4S] cluster of FadH is deeply buried in the structure and strongly liganded by four cysteine residues. Furthermore, the cluster appears to be very resistant to oxidation since it was preserved during FadH purification and crystallization (13). Moreover, a bacterial-like enzyme homologous to FadH has been found in eukaryotes such as Leishmania, indicative of a lateral transfer, and confirming the equivalence of the reactions performed by the enzymes of the two families (29). Further phylogenomic and enzymatic analyses of the eukaryotic DECR family might possibly provide a rationale for the emergence of [Fe-S]-less euDECR. Another intriguing potential twist in the evolution of these reductase families is provided by an enzyme of *Helicobacter pylori*, FabX, which possesses FMN and [4Fe-4S] cofactors like FadH, yet is required for the synthesis of unsaturated FAs and not for their degradation (30). Here too, it would be interesting to delineate the phylogenetic relationships between FadH and FabX enzymes.

In conclusion, our results show that it is crucial to better understand the degradation of various types of FAs, how these FAs impose different constraints on cell metabolism, whether prokaryotes or eukaryotes, which, in turn, will be differentially impacted by starvation or other types of stresses in the natural environment. In particular, we showed that enzymes of distinct protein families in bacteria or eukaryotes are able to perform the same 2,4-dienoyl-CoA reductase reaction *in vivo* in *E. coli*. This opens new lines of research as it suggests that in *E. coli*, an isomerase activity distinct from FadH is present to enable conversion from 3-*trans*-enoyl-CoA produced by euDECR to 2-*trans*-enoyl-CoA. Moreover, *E. coli fadH* mutant may provide a practical and powerful tool to study the structure/function of the eukaryotic enzymes important for human health.

## MATERIALS AND METHODS

### Media and growth conditions

Bacterial strains were routinely grown in aeration at 37°C in Luria-Bertani broth (LB; bactotryptone [10 g/liter], yeast extract [5 g/liter], NaCl [10 g/liter], pH 7.5) or M9 minimal medium (Na_2_HPO4-7H_2_O [6 g/liter], KH_2_PO_4_ [3 g/liter], NaCl [0.5 g/liter], NH_4_Cl [1 g/liter], MgSO_4_ [2 mM]), supplemented with 1.5% agar for solid media. When required, ampicillin was added at a concentration of 100 µg/mL. Growth on FAs was tested in synthetic M9 medium containing 1 g/L of potassium oleate or sodium linoleate. Potassium oleate 98% and sodium linoleate 95% were purchased from TCI. Petroselinic acid sodium salt was purchased from Santa Cruz Biotechnology. MCFAs and other oleate stocks were purchased from Sigma. All FAs were prepared at 100 mg/mL in 10% NP-40 and then diluted to a final concentration of 1 mg/mL in the culture media.

For growth assay on agar plates, after overnight growth in LB, cells were washed in minimal medium and normalized at an OD_600_ of 1, serially diluted, and spotted on M9 minimal medium containing ampicillin and supplemented with the carbon source to be tested (FAs, succinate, or glycerol). Plates were incubated for 3 days at 37°C. The agar plates shown for each experiment are representative of the results obtained in at least three independent experiments.

For liquid growth assays, cultures were started in LB in 96 deep-well plates, incubated for 6 hours at 37°C, and then diluted 200× in 800 µL of M9 minimal medium containing ampicillin and supplemented with the carbon source to be tested. After 48 hours at 37°C, 150 µL was transferred to a 96-well plate, and OD_600_ was measured using a Spark TECAN microplate reader. Note that the OD_600_ measured this way is not directly comparable with OD_600_ measured in a regular spectrophotometer and is also affected by the presence of the FAs. Only myristate precluded the direct measure of OD_600_ and in this case, we measured colony-forming units by serial dilutions and counting colonies after 1 day at 37°C on LB plates.

### Strain and plasmid constructions

The *E. coli* strains, plasmids, and oligonucleotides used in this study are listed, respectively, in Tables 1 and 2 and Table S1.

**TABLE 1:** Strains

Lab code	Name	Description	Reference
FBE353	MG1655	Wild-type reference strain	Lab collection
FBE765	∆*fadH*	MG1655 ∆*fadH*::kana^R^	This work
FBE189	*∆fadR*	MG1655 ∆*fadR*	This work
FBE1197	∆*fadH∆fadR*	MG1655 ∆*fadH* ∆*fadR*	This work
FBE434	∆*fadL*	MG1655 ∆*fadL*::kana^R^	This work
FBE425	∆*fadD*	MG1655 ∆*fadD*::kana^R^	This work
VSS50	∆*fadH∆fadR∆fadL*	MG1655 ∆*fadH* ∆*fadR* ∆*fadL*::kana^R^	This work

**TABLE 2:** Plasmids

Lab code	Name	Description	Reference
pEB266	pCP20	amp^R^, pTac promoter, *lacI*^q^	(31)
pVP114	pTrc99a	amp^R^, pTac promoter, *lacI*^q^	(32)
pEB1823	pTet	amp^R^, pTet promoter	(33)
pEB794	pJL148	amp^r^, kana^r^, SPA tag cassette	(34)
pVP037	pTet-SPA	SPA insert from pJL148 (NcoI/EcoRV) into pTet (NcoI/EcoRV)	This work
pVP589	pTrc-SPA	SPA insert from pVP037 (EcoRI/HindIII) in pTrc (EcoRI/HindIII) followed by mutagenesis with Ebp679/680 primers.	This work
pVP234	pTrc-*fadH*	PCR Ebm1742/1743 on *E. coli* genomic DNA (EcoRI/XhoI) in pTrc99a (EcoRI/SalI)	This work
pVP241	pTrc-*fadH*(C335A)	Mutagenesis PCR Ebp335/336 on pVP234	This work
pVP265	pTrc-*fadH*(C338A)	Mutagenesis PCR Ebp383/384 on pVP234	This work
pVP243	pTrc-*fadH*(C342A)	Mutagenesis PCR Ebp339/340 on pVP234	This work
pVP244	pTrc-*fadH*(C354A)	Mutagenesis PCR Ebp341/342 on pVP234	This work
pVP240	pBAD-6His-DECR2_human_cDNA		(20)
pVP247	pTrc-DECR2_human_cDNA	PCR Ebp348/349 on pVP240 (EcoRI/XhoI) in pTrc99a (EcoRI/SalI)	This work
pVP332	pTrc-DECR1∆TP_human	PCR Ebp449/424 on synthetic fragment (EcoRI/XhoI) in pTrc99a (EcoRI/SalI)	This work
pVP333	pTrc-DECR1∆TP_rat	Ebp450/428 on synthetic fragment (EcoRI/XhoI) in pTrc99a (EcoRI/SalI)	This work
pVP318	pTrc-DECR2_rat	Ebp429/430 on synthetic fragment (EcoRI/XhoI) in pTrc99a (EcoRI/SalI)	This work
pVP394	pTrc-*fadD*	PCR Ebm302/303 on *E. coli* genomic DNA (EcoRI/XhoI) in pTrc99a (EcoRI/SalI)	This work
pVP400	pTrc-*fadD*(Y213A)	Mutagenesis PCR ebp516/517 on pVP394	This work
pVP401	pTrc-*fadD*(E361A)	Mutagenesis PCR ebp518/519 on pVP394	This work
pVP395	pTrc-*fadB*	PCR Ebm1731/1732 on *E. coli* genomic DNA (EcoRI/XhoI) in pTrc99a (EcoRI/SalI)	This work
pVP396	pTrc-*fadE*	PCR Ebm1740/1741 on *E. coli* genomic DNA (EcoRI/XhoI) in pTrc99a (EcoRI/SalI)	This work
pVP397	pTrc-*fadBA*	PCR Ebm1731/1725 on *E. coli* genomic DNA (EcoRI/XhoI) in pTrc99a (EcoRI/SalI)	This work
pVP398	pTrc-*fadIJ*	PCR Ebm1733/305 on *E. coli* genomic DNA EcoRI/HindIII in pTrc99a (EcoRI/HindIII)	This work
pVP569	pTrc-*fadL*	PCR Ebp676/162 on *E. coli* genomic DNA EcoRI/XhoI in pTrc99a (EcoRI/XhoI)	This work
pVP581	pTrc-*fadH-SPA*	PCR Ebm1742/1743N on *E. coli* genomic DNA (EcoRI/NcoI) in pTrc-SPA (EcoRI/NcoI)	This work
pVP566	pTrc-*fadD-SPA*	PCR Ebm302/303N on *E. coli* genomic DNA (EcoRI/NcoI) in pTrc-SPA (EcoRI/NcoI)	This work
pVP586	pTrc-*DECR2h-SPA*	PCR Ebp348/349N on pVP240 (EcoRI/NcoI) in pTrc-SPA (EcoRI/NcoI)	This work
pVP595	pTrc-DECR1h-SPA	PCR Ebp449/681 on synthetic fragment (EcoRI/NcoI) in pTrc-SPA (EcoRI/NcoI)	This work
pVP596	pTrc-*DECR2r-SPA*	PCR Ebp688/689 on synthetic fragment (EcoRI/BsphI) in pTrc-SPA (EcoRI/NcoI)	This work
pVP597	pTrc-*DECR1r-SPA*	PCR Ebp450/683 on synthetic fragment (EcoRI/NcoI) in pTrc-SPA (EcoRI/NcoI)	This work

The ∆*fadH*, ∆*fadL*, ∆*fadR*, and ∆*fadD E. coli* deletion mutants were constructed by P1 transduction from the corresponding strains of the Keio collection (35) into our laboratory’s MG1655 reference strain. The kanamycin resistance cassette was then removed by specific recombination using the pCP20 plasmid (31) as described previously (36). The double and triple mutants were constructed similarly by successive transductions and excisions of the kanamycin resistance cassette.

For plasmid construction, DNA fragments were amplified by PCR and cloned into pTrc99a (pVP114) (32) or pTrc-SPA (pVP589) plasmids using conventional cloning with restriction enzymes and T4 DNA ligase. The pTrc-SPA plasmid was obtained in successive steps: transfer of the SPA sequence from pJL148 to pTet plasmid (33) using NcoI/EcoRV restriction sites; transfer of the cloning sites and SPA sequence from this pTet-SPA plasmid (pVP037) in pTrc99a using EcoRI/HindIII restriction sites; and finally destroying an NcoI restriction site using directed mutagenesis. The primers and restriction enzymes used for constructing each plasmid are given in Table 2. The coding sequences of DECR1, deleted of the N-terminal targeting peptide, and of rat DECR2 have been codon-optimized for expression in *E. coli* (Table S2), and genes have been ordered from Twist Bioscience (TWB). Additionally, the natural coding sequence of human DECR2 has been amplified from the pBAD-DECR2 plasmid (20). Mutations were introduced in the pTrc-*fadH*, pTrc-*fadD*, pTrc-DECR2h, pTrc-*fadH-*SPA, pTrc-*fadD-*SPA, and pTrc-DECR2h-SPA plasmids by PCR mutagenesis using the oligonucleotides indicated (Table 2; Table S1).

Spontaneous suppressors of the ∆*fadH*∆*fadR* strain were first isolated by plating approximately 10^8^ cells on an M9 plate containing 0.1% linoleate and 0.1% decanoate. Clones were obtained at a frequency of about 3.10^−6^. The colonies were reisolated on nonselective LB plates and then tested again for growth on linoleate + decanoate as shown in Fig. 4A. We then amplified the *fadL* locus by PCR with primers ebp500 and ebp162 and sequenced the product.

To avoid suppressor mutations in the *fadL* locus, we then performed a second selection, this time on linoleate and myristate, following the same procedure as described above. The genomic DNA of several suppressors was prepared using the Monarch Genomic DNA purification kit from NEB and sent for whole-genome sequencing with the INVIEW resequencing service at Eurofins.

### Sodium dodecyl sulfate-polyacrylamide gel electrophoresis and Western blot to detect tagged proteins

Wild-type *E. coli* transformed by the different pTrc or pTrc-SPA plasmids were grown in LB supplemented with ampicillin at 37°C. At OD_600_ = 1, 1 mL culture was centrifuged, and total extracts were prepared by resuspending cell pellets in 1X Laemmli Loading buffer (30 µL/OD_600_ = 1) and heating 10 minutes at 95°C. When needed, the expression was induced with 1 mM IPTG for 2 hours, and total extracts were prepared similarly. SDS-PAGE and Western blot were performed as previously described (33). The SPA-tagged proteins were detected using the monoclonal anti-Flag antibody. The polyclonal anti-YbgF (CpoB) antibody was used as an internal control. Fluorescent secondary antibodies were, respectively, IRDye 800 anti-mouse and IRDye 680 anti-rabbit purchased from Li-Cor. Scanning was performed on a Li-Cor Odyssey-Fc imaging system reading at 700 nm (for YbgF detection, colored in red in the figures) or 800 nm (for Flag detection, colored in green in the figures).

### Lipid extraction and analysis by TLC and normal phase chromatography coupled to mass spectrometry

Wild-type and mutant strains were grown in 100 ml M9 minimal medium containing 0.1% oleate (O) as the sole carbon source at 37°C. At OD_600_ = 1.5 (2 days of growth), cells were washed and resuspended in 100 mL M9 minimal medium containing 0.1% oleate (O) or 0.1% oleate plus 0.1% linoleate (O + L) as the sole carbon source, either at OD_600_ = 0.1 for wild-type and complemented ∆*fadH* strains, or with no dilution for the ∆*fadH* strain. After two more days at 37°C (all cultures at approximately OD_600_ = 1.5), cells were pelleted and washed twice with phosphate-buffered saline. Total lipid extraction was performed through a neutral Bligh-Dyer method (37), following the protocol detailed in reference 38.

Lipids were analyzed by TLC using a solvent for separation of neutral lipids (heptane, ether, formic acid 55:45:1). We used a commercial mono-, di-, and triglyceride standard mix (Humeau, #226542), and as a reference for FAs, we used lipid extract from a ∆*fadD* strain, which has been shown to accumulate free FAs (39). After drying the plate, lipids were visualized by spraying with primuline, and the picture was taken under UV exposure.

FAs present in the same extracts were identified using a previously described method (40). Briefly, lipid analysis was performed by coupling a Dionex-U3000 quaternary RSLC liquid chromatograph to an LTQ-Orbitrap Velos Pro hybrid orbital trap mass spectrometer (ThermoFisher Scientific). Lipid extracts of the three strains were solubilized in 100 µL of chloroform, and 5 µL was injected. Lipid separation was performed by high-performance liquid chromatography (HPLC) on a U3000 system using an Inertsil Si 5 µm column (150 × 2.1 mm I.D.), normal phase stationary phase from GL Sciences Inc (Tokyo, Japan). FAs were identified regarding their retention time and by high-resolution mass spectrometry negative atmospheric pressure ionization (LTQ Orbitrap Velos Pro).

## SUPPLEMENTAL MATERIAL

Supplemental material mbio.02203-25-s0001.pdf Tables S1 and S2; Figures S1 to S6.

ASM does not own the copyrights to Supplemental Material that may be linked to, or accessed through, an article. The authors have granted ASM a non-exclusive, world-wide license to publish the Supplemental Material files. Please contact the corresponding author directly for reuse.
