# Supplementary material for: The essential role of 2,4-dienoyl-CoA reductase for the degradation of complex fatty acid mixtures
Source: mBio. 2025 Oct 29;16(12):e02203-25. doi: 10.1128/mbio.02203-25 (PMC12691678; doi:10.1128/mbio.02203-25)
Supplement: Supplemental material — Tables S1 and S2; Figures S1 to S6. [file mbio.02203-25-s0001.pdf]

Supplementary Materials for

The essential role of 2,4-dienoyl-CoA reductase for degradation of complex fatty acid mixtures

Veronica Schiaffi*1, Viola Pavoncello*1, Bastien Prost2, Audrey Solgadi2, Frédéric Barras1, Emmanuelle Bouveret1#

This file includes :

- Tables S1 and S2
- Figures S1 to S6

Table S1 : primers

Lab code	Sequence	Name
Ebm302	caccgaattcttgAAGAAGGTTTGGCTTAACCG	fadD FW
Ebm303	ctactcgagaagcttaGGCTTTATTGTCCACTTTGCCG	fadD RV
Ebm1742	TGGGAATTCatgAGCTACCCGTCGCTGTTC	fadH FW
Ebm1743	ccgctcgagttaAATCTCCAGCGCCAGCC	fadH RV
Ebm1740	TGGGAATTCatgATGATTTTGAGTATTCTCGCTACG	fadE FW
Ebm1741	ccgctcgagttaCGCGGCTTCAACTTTCCG	fadE RV
Ebm1731	TGGGAATTCATGCTTTACAAAGGCGACACCC	fadB FW
Ebm1732	ccgctcgagttaAGCCGTTTTCAGGTCGCC	fadB RV
Ebm1725	ccgctcgagTTAAACCCGCTCAAACACCGTCG	fadA RV
Ebm1733	TGGGAATTCatgGGTCAGGTTTTACCGCTGG	fadI FW
Ebm305	ctactcgagaagcttaTTGCAGGTCAGTTGCAGTTGTTTTC	fadJ RV
Ebp676	gctGAATTCatgGTCATGAGCCAGAAAACCC	fadL FW
Ebp162	acgctcgagtcaGAACGCGTAGTTAAAGTTAGTAC	fadL RV
Ebp335	GATGAGATCAACACTGCTATTGGCTGCAATCAG	fadH C335A FW
Ebp336	CTGATTGCAGCCAATAGCAGTGTTGATCTCATC	fadH C335A RV
Ebp383	AACACTTGTATTGGCGCCAATCAGGCCTGTCTC	fadH C338A FW
Ebp384	GAGACAGGCCTGATTGGCGCCAATACAAGTGTT	fadH C338A RV
Ebp339	GGCTGCAATCAGGCCGCTCTCGATCAAATCTTC	fadH C342A FW
Ebp340	GAAGATTTGATCGAGAGCGGCCTGATTGCAGCC	fadH C342A RV
Ebp341	GGCAAAGTCACCTCGGCCCTGGTGAATCCTCGC	fadH C354A FW
Ebp342	GCGAGGATTCACCAGGGCCGAGGTGACTTTGCC	fadH C354A RV
Ebp348	actGAATTCATGGCCCAGCCGCC	DECR2h cDNA FW
Ebp349	acgctcgagTTAGAGCTTAGCAGAGAAGGATGC	DECR2h cDNA RV
Ebp449	actGAATTCATGAATACCGAGGCATTACAGTCGAAG	DECR1h FW
Ebp424	acgctcgagTTAGCTGCCCTTGGTTTTGC	DECR1h RV
Ebp425	actGAATTCATGGCTCAGCCGCCCC	DECR2h FW
Ebp426	acgctcgagTTACAGTTTTGCACTAAAAGAAGC	DECR2h RV
Ebp450	actGAATTCATGTCTATTGACGCCCCTCAATCC	DECR1r FW
Ebp428	acgctcgagTTACGAACCCTTAGTTTTACGAATC	DECR1r RV
Ebp429	actGAATTCATGACGCAACAACCACCCG	DECR2r FW
Ebp430	acgctcgagTCAAAGTTTCGCGGATGATGACTC	DECR2r RV

Ebp516	acgctcgagTCAAAGTTTCGCGGATGATGACTC	fadD Y213A FW
Ebp517	acgctcgagTCAAAGTTTCGCGGATGATGACTC	fadD Y213A RV
Ebp518	acgctcgagTCAAAGTTTCGCGGATGATGACTC	fadD E361A FW
Ebp519	acgctcgagTCAAAGTTTCGCGGATGATGACTC	fadD E361A RV
Ebp357	CTCCCTCTCTCTATGGCCGTCCGAGCGCC	DECR2 D86A FW
Ebp358	GGCGCTCGGACGGCCATAGAGAGAGGGAG	DECR2 D86A RV
Ebp359	CGTGATGGACATCGCGACCAGCGGCACC	DECR2 D137A FW
Ebp360	GGTGCCGCTGGTCGCGATGTCCATCACG	DECR2 D137A RV
Ebp412	ggccgctgtggccgcgatgacgc	DECR2 D186A FW
Ebp413	gcgtcatcgcggccacagcggcc	DECR2 D186A RV
Ebp414	gctggtggccgctggcggggcat	DECR2 D268A FW
Ebp414b	atgccccgccagcggccaccagc	DECR2 D268A RV
Ebp419	ctgtgcggccggggccttcctgtgcccc	DECR2 N117A FW
Ebp420	ggggcacaggaaggccccggccgcacag	DECR2 N117A RV
Ebp421	tgcaggctccgccgcggccgctgtggac	DECR2 K182A FW
Ebp422	gtccacagcggccgcggcggagcctgca	DECR2 K182A RV
Ebp162	acgctcgagtcaGAACGCGTAGTTAAAGTTAGTAC	DOWN *fadL* RV
Ebp500	cgggatccCTCCAAATTTTGCCAGCTGGATC	Up *fadL* FW
Ebp679	ttcacacaggaaacagaGcatggaattcgagctcg	pTrc-SPA mutNcoI FW
Ebp680	cgagctcgaattccatgCtctgtttcctgtgtgaa	pTrc-SPA mutNcoI RV
Ebp681	tttccatggtGCTGCCCTTGGTTTTGCG	DECR1h RV
Ebp683	tttccatggtCGAACCCTTAGTTTTACGAATCAG	DECR1r RV
Ebp688	ACTTCATGAtAAGTTTCGCGGATGATGACTC	DECR2r FW
Ebp689	actGAATTCgATGACGCAACAACCACCCG	DECR2r RV
Ebp349N	tttccatggtGAGCTTAGCAGAGAAGGATGC	DECR2h RV
Ebm1743N	tttccatggtAATCTCCAGCGCCAGCC	fadH RV
Ebm303N	tttccatggtGGCTTTATTGTCCACTTTGCCG	fadD RV

Table S2 : DNA sequences of the cloned eukaryotic DECR enzymes. The EcoRI and XhoI restriction sites appended to the sequences for plasmid constructions are indicated in bold.

**DECR2 human c_DNA**<br>**GAATTC**ATGGCCCAGCCGCCGCCCGACGTGGAGGGGGACGACTGTCTCCCCGCGTACCGCCACCTCTTCTGCC<br>CGGACCTGCTGCGGGACAAAGTGGCCTTCATCACAGGAGGCGGCTCTGGGATTGGGTTCCGGATTGCTGAGAT<br>TTTCATGCGGCACGGCTGCCATACGGTGATTGCCAGTAGGAGCCTGCCGCGAGTGCTGACGGCCGCCAGGAAG<br>CTGGCTGGGGCCACCGGCCGGCGCTGCCTCCCTCTCTCTATGGACGTCCGAGCGCCCCCAGCTGTCATGGCCG<br>CCGTGGACCAGGCTCTGAAGGAGTTTGGCAGAATCGACATTCTCATTAACTGTGCGGCCGGGAACTTCCTGTG<br>CCCCGCTGGCGCCTTGTCCTTCAACGCCTTCAAGACCGTGATGGACATCGATACCAGCGGCACCTTCAATGTG<br>TCTCGTGTGCTCTATGAGAAGTTCTTCCGGGACCACGGAGGGGTGATCGTGAACATCACTGCCACCCTGGGGA<br>ACCGGGGGCAGGCGCTCCAGGTGCATGCAGGCTCCGCCAAGGCCGCTGTGGACGCGATGACGCGGCACTTGGC<br>TGTGGAGTGGGGTCCCCAAAACATCCGCGTCAACAGCCTCGCCCCTGGCCCCATCAGTGGCACAGAGGGGCTC<br>CGGCGACTGGGTGGCCCTCAGGCCAGCCTGAGCACCAAGGTCACTGCCAGCCCGCTGCAGAGGCTGGGGAACA<br>AGACCGAGATCGCCCACAGCGTGCTCTACCTGGCCAGCCCTCTGGCTTCCTACGTGACGGGGGCCGTGCTGGT<br>GGCCGATGGCGGGGCATGGTTGACGTTCCCAAACGGTGTCAAAGGGCTGCCGGATTTCGCATCCTTCTCTGCT<br>AAGCTCTAA**CTCGAG**
**DECR2 rat optimized**<br>**GAATTC**ATGACGCAACAACCACCCGATGTGGAAGAAGACGATTGCCTGTCCGAGTATCATCATTTATTTTGCC<br>CAGATCTGTTGCAAGATAAGGTTGCATTCATAACGGGAGGAGGCTCCGGTATAGGTTTTCGCATTGCAGAAAT<br>CTTTATGCGCCACGGTTGTCATACGGTAATAGTTAGTAGATCTTTACCCCGTGTTTCAGAAGCAGCAAAGAAA<br>CTCGTGGCAGCGACCGGGAAACGCTGCTTGCCCTTATCCATGGACGTGCGCGTCCCACCCGCCGTTATGGCAG<br>CAGTCGATCAGGCATTAAAGGAGTTCGGGAAGATTGATATTTTGATCAATTGCGCAGCGGGTAATTTCCTTTG<br>TCCAGCATCGGCCCTGTCATTTAACGCGTTCAAAACGGTTGTTGATATCGATACTCTGGGTACATTTAACGTC<br>AGCCGGGTACTGTACGAGAAATTCTTTCGCGATCACGGCGGCGTCATTGTCAATATCACGGCTACGCTGTCAA<br>TGCGTGGCCAAGTCCTTCAACTTCACGCGGGTGCCGCAAAAGCTGCGGTAGACGCAATGACTCGTCATCTGGC<br>GGTAGAATGGGGTCCGCAAAACATTCGCGTGAATAGTCTTGCGCCAGGCGCAATATCCGGAACAGAAGGGTTG<br>CGACGTTTGGGTGGACCAAAAGCATCCTCTAAGTTCAAATACCTGTCCTCTCCGATCCCGCGACTGGGGACTA<br>AAACTGAGATTGCTCATAGTGTATTATATCTGGCGTCTCCGTTAGCAAGTTACGTAAGTGGAATCGTATTAGT<br>AGTAGACGGTGGCAGTTGGATGACATTACCGAACGATATCGGACGTCTCCTGGAATTTGAGTCATCATCCGCG<br>AAACTTTGA**CTCGAG**
**DECR1 ΔTarget Peptide human optimized**<br>**GAATTC**AATACCGAGGCATTACAGTCGAAGTTCTTCAGTCCGTTACAGAAGGCCATGCTTCCCCCGAACTCTT<br>TCCAGGGCAAGGTCGCTTTTATAACCGGCGGTGGAACCGGACTGGGCAAGGGTATGACCACACTGTTAAGTAG<br>TCTGGGCGCCCAATGTGTTATTGCATCGCGCAAAATGGACGTCTTAAAGGCAACGGCCGAGCAGATCAGCAGC<br>CAGACCGGTAACAAAGTGCACGCGATACAATGCGACGTCCGCGACCCGGACATGGTACAGAATACCGTTTCTG<br>AGCTTATTAAGGTAGCGGGCCACCCGAACATAGTAATCAATAACGCGGCTGGTAACTTCATCTCCCCAACAGA<br>GCGACTCAGCCCCAACGCGTGGAAGACTATTACGGATATTGTGTTAAACGGAACTGCGTTTGTCACTTTAGAG<br>ATCGGCAAGCAGCTGATCAAGGCTCAAAAGGGCGCCGCTTTCTTGTCAATCACCACCATTTACGCCGAAACCG<br>GCAGCGGATTCGTGGTGCCGTCTGCATCGGCGAAAGCCGGGGTCGAGGCAATGTCAAAATCGTTGGCGGCGGA<br>GTGGGGCAAGTACGGCATGAGATTTAACGTTATACAGCCGGGACCGATTAAGACTAAGGGAGCATTCTCCAGA<br>CTTGATCCCACCGGTACGTTCGAAAAGGAGATGATAGGTCGCATACCTTGCGGGCGTTTGGGAACCGTTGAGG<br>AGTTAGCTAACCTGGCCGCGTTTCTGTGCTCTGACTACGCGAGCTGGATAAACGGCGCCGTAATCAAGTTCGA<br>TGGCGGGGAAGAGGTGCTGATCAGCGGCGAGTTTAATGATTTGCGGAAAGTTACGAAAGAACAATGGGATACG<br>ATTGAGGAGCTTATTCGCAAAACCAAGGGCAGCTAA**CTCGAG**
**DECR1 ΔTarget Peptide rat optimized**<br>**GAATTC**TCTATTGACGCCCCTCAATCCAAGTTCTTCCCGCCAATACTGAAACCCATGTTACCGCCCAACGCAT<br>TCCAGGGCAAGGTTGCATTTATTACAGGTGGCGGTACCGGGCTCGGGAAAGCCATGACCACCTTTAGAAGTAG<br>TCTCGGGGCTCAATGCGTTATAGCATCCCGCAACATCGACGTGCTTAAGGCCACCGCTGAGGAAATCACCAGT<br>AAGACAGGTAACAAAGTTTACGCCATACGTTGCGATGTCCGTGACCCAGACATGGTTCATAATACGGTCTTGG<br>AACTTATTAAGGTGGCGGGCCACCCGGACGTTGTCATCAATAACGCCGCCGGCAATTTTATATCGCCGTCCGA<br>ACGCTTAAGCCCAAACGGCTGGCGAACAATCACCGATATCGTACTGAACGGAACTGCTTACGTCACAATCGAG<br>ATCGGTAAACAACTGATCAAGGCGCAAAAGGGCGTGGCATTCTTGGCCATTACAACCATTTACGCCGAATCGG<br>GTAGTGGTTTCGTCATGCCGTCCAGCTCTGCTAAGAGCGGGGTCGAGGCTATGAACAAAAGTTTAGCCGCCGA<br>GTGGGGCCGCTATGGTATGCGATTTAATATTATACAACCCGGTCCAATTAAGACTAAGGGCGCTTTCTCGCGG<br>TTAGATCCTACAGGCAAGTTCGAAAAGGACATGATTGAACGTATTCCGTGCGGGCGTTTAGGTACAGTTGAAG<br>AGTTGGCTAACCTTGCCACATTTCTGTGTTCCGACTACGCGAGCTGGATTAACGGAGCCGTTATCAGATTCGA<br>TGGCGGGGAAGAGGTTTTCCTTTCCGGCGAGTTTAATTCACTGAAGAAGGTGACGAAAGAAGAATGGGACGTG<br>ATAGAGGGTCTGATTCGTAAAACTAAGGGTTCGTAA**CTCGAG**

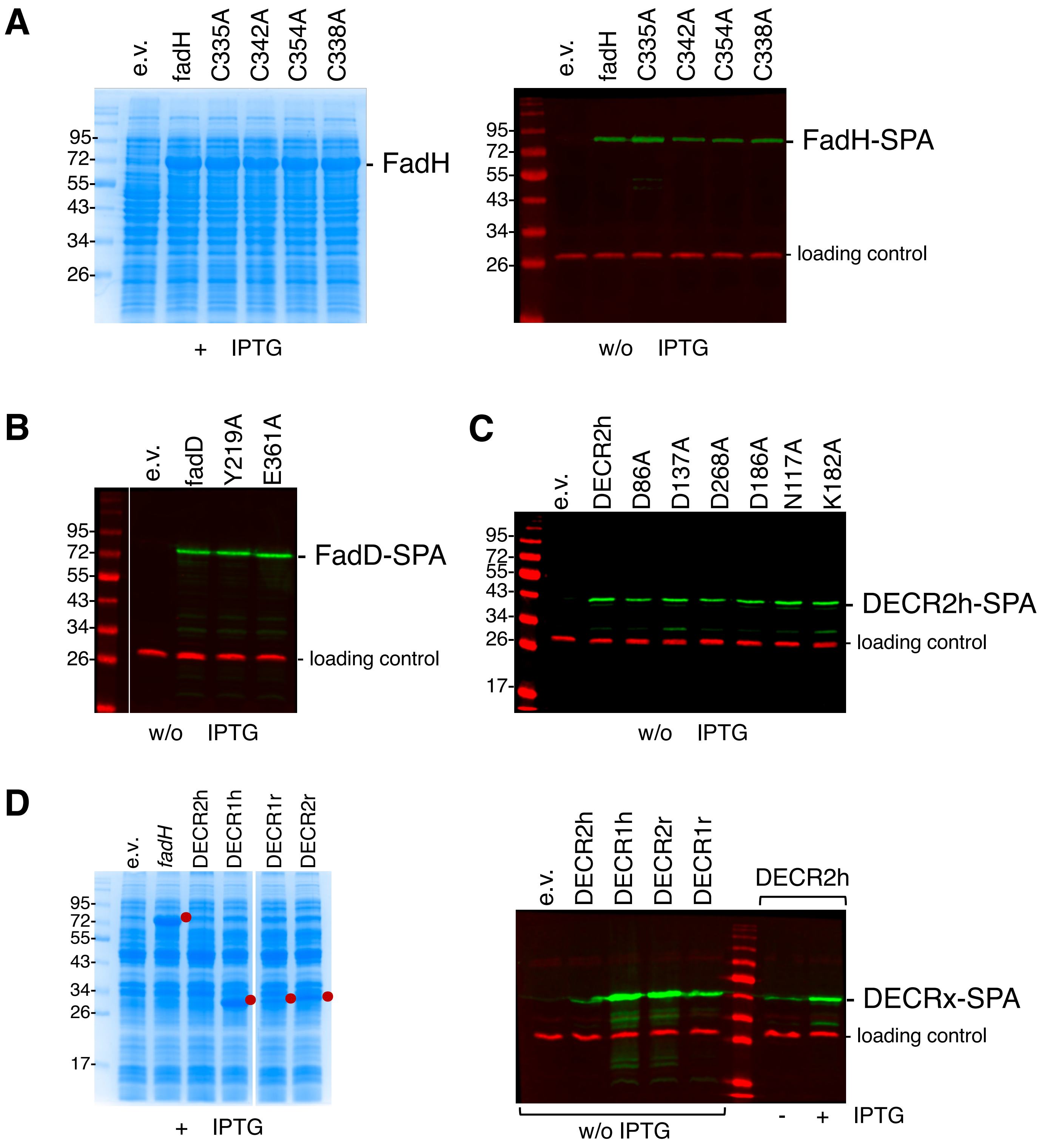

Figure S1 : **A.** Wild-type *E. coli* strain was transformed by the pTrc and pTrc-SPA plasmids containing the indicated FadH mutants. Production of the proteins was then assayed by detecting overproduction after 1 mM IPTG induction (left panel) or without (w/o) induction by Western blot (right panel) as described in Materials and Methods. **B.C.** Wild-type *E. coli* strain was transformed by the pTrc-FadD-SPA **(B)** and pTrc-DECR2h-SPA **(C)** plasmids bearing the indicated mutations. Production of the proteins, without induction, was then assayed by Western blot as described in Materials and Methods. **D.** Wild-type *E. coli* strain was transformed by the pTrc (left panel) and pTrc-SPA (right panel) plasmids containing the indicated DECRs genes. Production of the proteins was then assayed by detecting overproduction after 1mM IPTG induction (left panel) or without (w/o) induction by Western blot (right panel) as described in Materials and Methods. A comparison with or without induction (-/+ IPTG) was also performed by western blot on the pTrc-DECR2h-SPA plasmid. On the Coomassie-blue stained gel, red dots indicate the position of the apparent overproduced proteins. The white separation indicates a cut in the image to show different parts of the same gel.

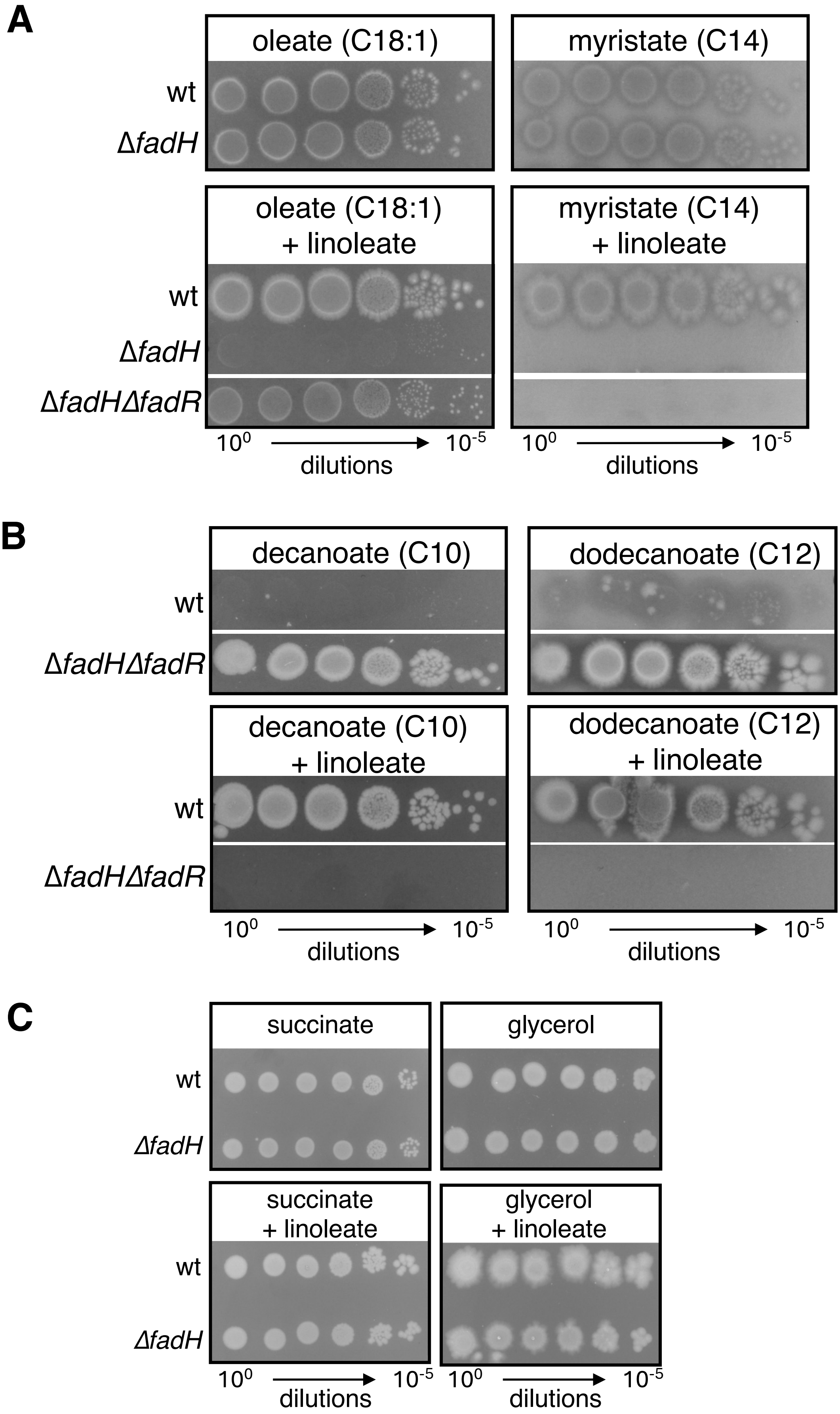

Figure S2 : A. Wild-type, *fadH* (FBE765), and Δ*fadH*Δ*fadR* (FBE1197) mutant strains were tested for growth on minimal medium containing 0.1% oleate or myristate, with or without the addition of 0.1% linoleate, as described in Materials and Methods. **B.** Wild type and Δ*fadH*Δ*fadR* mutant (FBE1197) strains were tested for growth on minimal medium containing 0.1% decanoate or dodecanoate, with or without the addition of 0.1% linoleate. **C.** Wild-type and Δ*fadH* mutant (FBE765) strains were tested for growth on minimal medium containing 0.2% glycerol or 0.4% succinate, with or without 0.1% linoleate. Each panel shows a picture taken from one plate; the white lines indicate a cut to show different parts of the same plate. Plates were incubated at 37°C for 3 days.

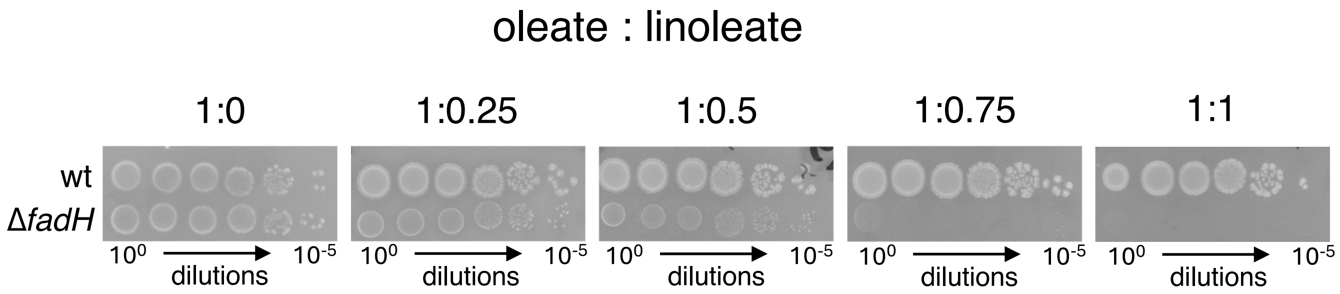

Figure S3 : Linoleate prevents consumption of oleate by the *fadH* mutant in proportion with its amount. After overnight growth in LB, wild-type and Δ*fadH* (FBE765) cells were washed in minimal medium, serially diluted, and spotted on M9 minimal medium plates containing 0.1% oleate and increasing amounts of linoleate from 0 (1:0) to 0.1% (1:1). Plates shown were incubated at 37°C for 3 days.

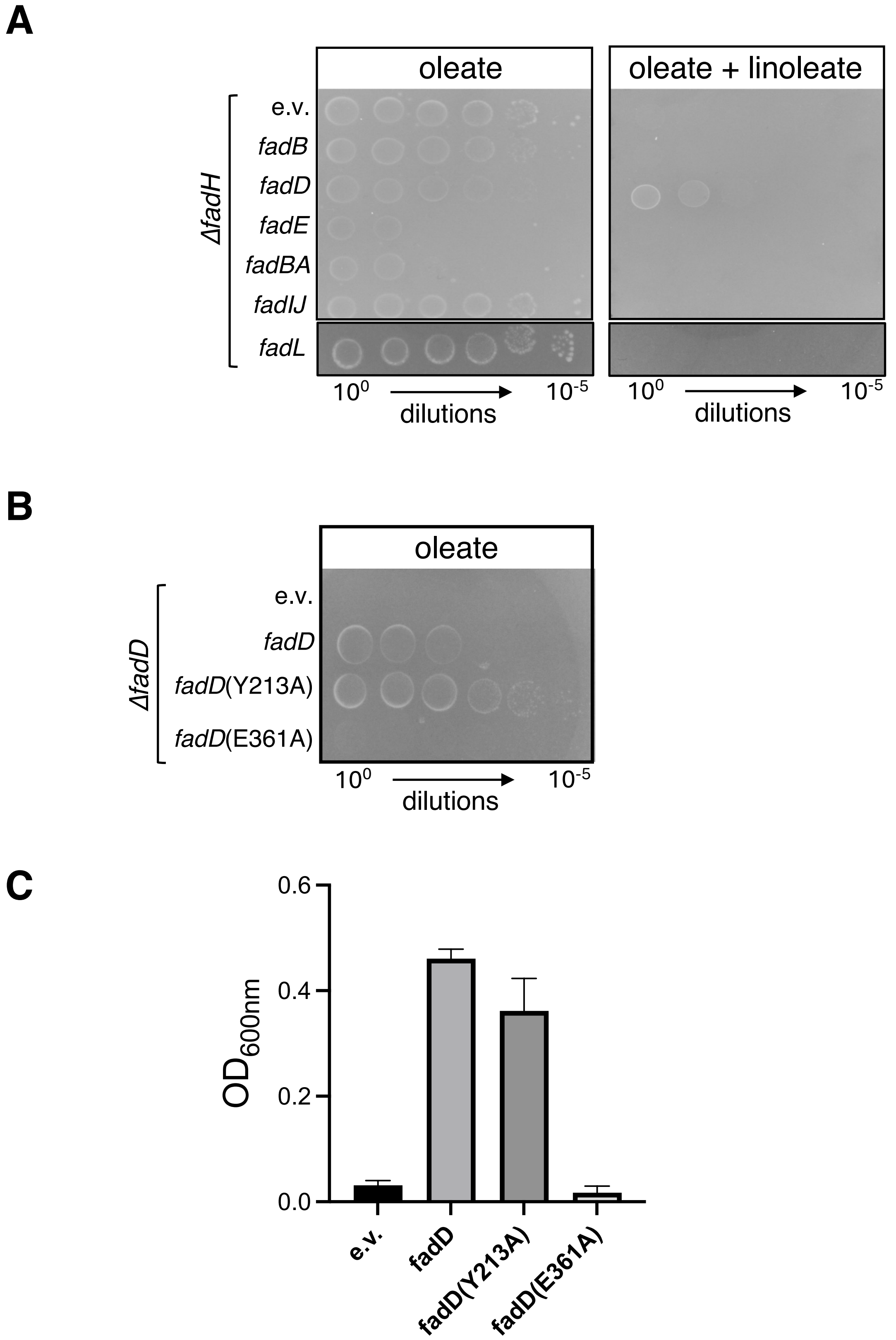

Figure S4 : A. The Δ*fadH* mutant strain (FBE765) was transformed by plasmids expressing the different *fad* genes as indicated. Growth was assayed on M9 minimal plates containing 0.1% oleate as the sole carbon source, with or without 0.1% linoleate, as described in Materials and Methods. **B.** The Δ*fadD* mutant strain (FBE425) was transformed by the indicated plasmids. Growth was assayed on M9 minimal plates containing 0.1% oleate as the sole carbon source, as described in Materials and Methods. **C.** The Δ*fadD* mutant strain (FBE425) was transformed by the indicated pTrc-*fadD*-SPA plasmids and assayed for liquid growth in M9 minimal medium containing 0.1% oleate as the sole carbon source.

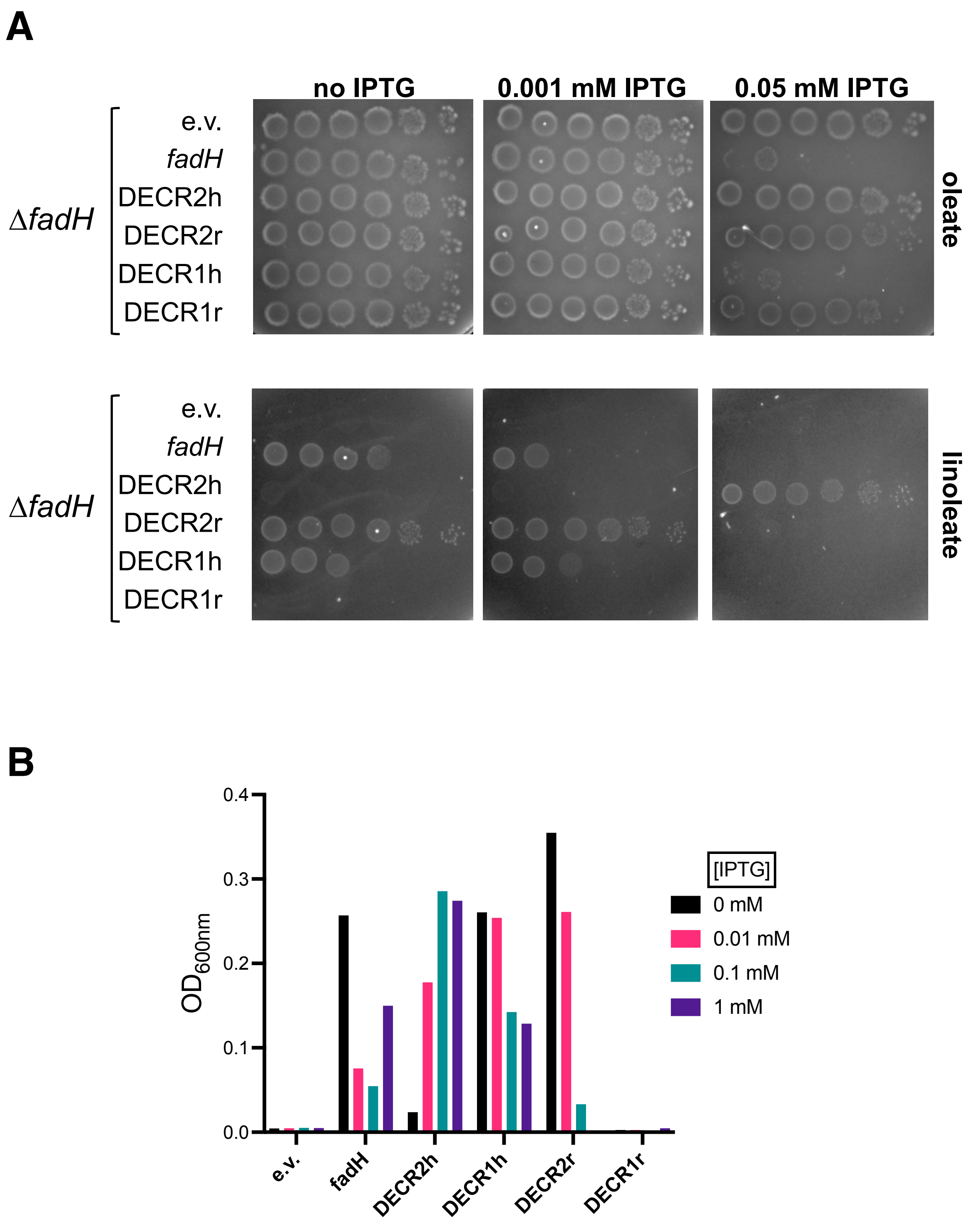

Figure S5 : A. The *fadH* deletion strain (FBE765) was transformed by the pTrc99a empty vector (e.v.), the *fadH* vector, or plasmids encoding the indicated DECR genes. The transformed strains were tested for growth on minimal medium containing oleate (upper panels) or linoleate (lower panels) containing the indicated concentration of IPTG as described in Materials and Methods. **B.** The same strains were tested for growth in liquid medium containing linoleate with different concentrations of IPTG as described in Materials and Methods.

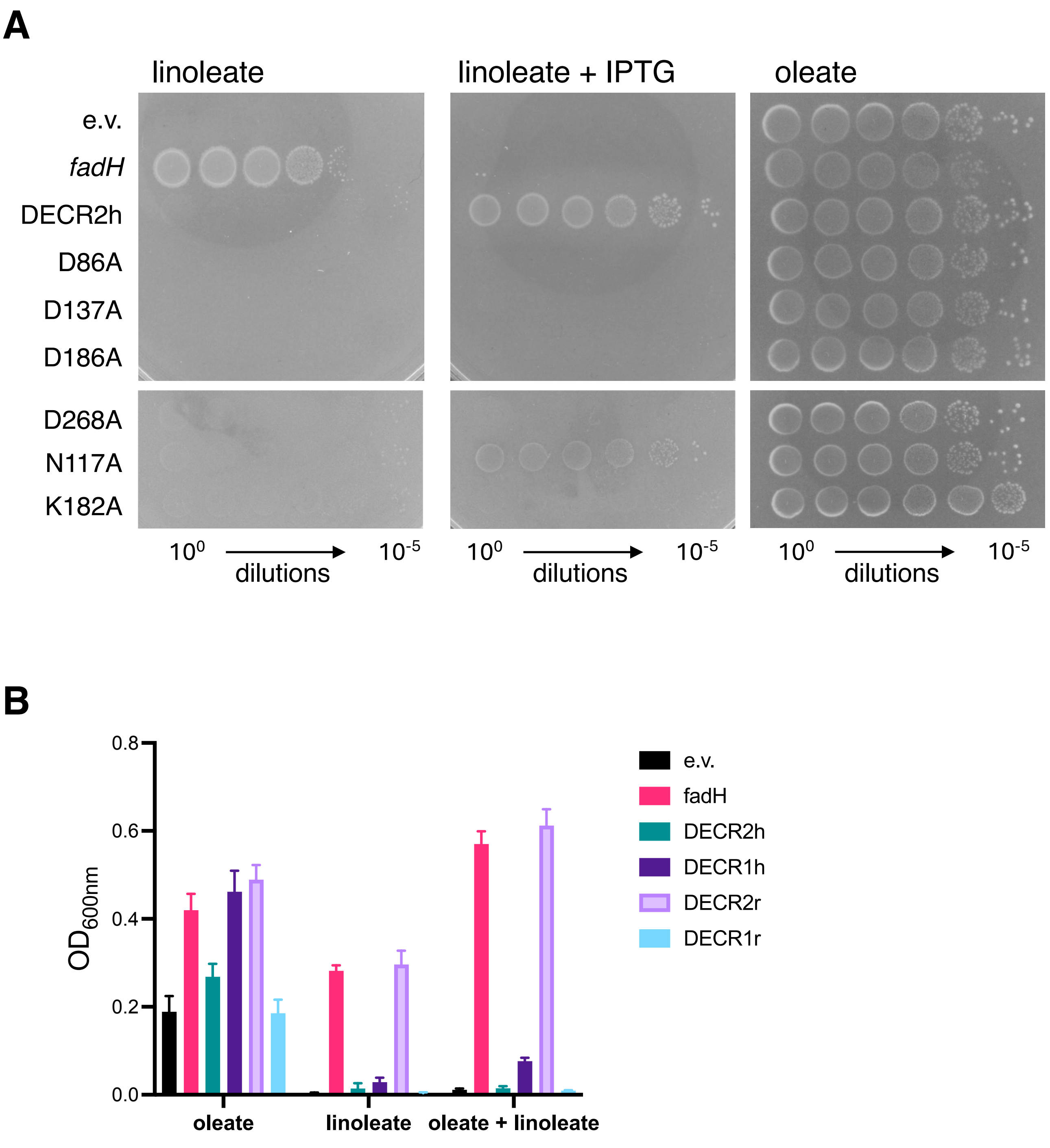

Figure S6 : **A.** The *fadH* deletion strain (FBE765) was transformed by the pTrc99a empty vector (e.v.), the *fadH* vector, or plasmids encoding the indicated mutated DECR2h genes. The transformed strains were tested for growth on minimal medium containing linoleate with or without 0.5 mM IPTG, or oleate as described in Materials and Methods. **B.** The *fadH* deletion strain (FBE765) was transformed by pTrc99a empty vector (e.v.), *fadH* vector, or plasmids encoding the indicated DECR genes. The transformed strains were tested for growth in liquid minimal medium containing oleate, linoleate, or oleate + linoleate as described in Materials and Methods.
